# Exercise-based rehabilitation on functionality and quality of life in head and neck cancer survivors. A systematic review and meta-analysis

**DOI:** 10.1038/s41598-023-35503-y

**Published:** 2023-05-26

**Authors:** Isidro Miguel Martín Pérez, Sebastián Eustaquio Martín Pérez, Raquel Pérez García, Diego de Zárate Lupgens, Germán Barrachina Martínez, Carolina Rodríguez González, Nart Keituqwa Yáñez, Fidel Rodríguez Hernández

**Affiliations:** 1 Departamento de Medicina Física y Farmacología, Área de Radiología y Medicina Física, Facultad de Ciencias de la Salud, Universidad de la Laguna, 38200 Santa Cruz de Tenerife, Spain; 2 Escuela de Doctorado y Estudios de Posgrado, Universidad de la Laguna, 38203 San Cristóbal de La Laguna, Santa Cruz de Tenerife, Spain; 3 Musculoskeletal Pain and Motor Control Research Group, Faculty of Health Sciences, Universidad Europea de Canarias, 38300 La Orotava, Santa Cruz de Tenerife, Spain; 4 Musculoskeletal Pain and Motor Control Research Group, Faculty of Sport Sciences, Universidad Europea de Madrid, 28670 Villaviciosa de Odón, Madrid, Spain; 5 Hospital Universitario de Canarias, 38320 San Cristóbal de la Laguna, Santa Cruz de Tenerife, Spain

**Keywords:** Surgical oncology, Oncology, Cancer, Head and neck cancer, Oral cancer

## Abstract

Head and Neck Cancer (HNC) is a globally rare cancer that includes a variety of tumors affecting the upper aerodigestive tract. It presents with difficulty breathing or swallowing and is mainly treated with radiation therapy, chemotherapy, or surgery for tumors that have spread locally or throughout the body. Alternatively, exercise can be used during cancer treatment to improve function, including pain relief, increase range of motion and muscle strength, and reduce cancer-related fatigue, thereby enhancing quality of life. Although existing evidence suggests the adjunctive use of exercise in other cancer types, no previous studies have examined the effects on HNC survivors. The aim of this meta-analysis was to quantify the effect of exercise-based rehabilitation on functionality and quality of life in HNC survivors who underwent surgery and/or chemoradiotherapy. A systematic review and meta-analysis were carried out following PRISMA statement and registered in PROSPERO (CRD42023390300). The search was performed in MEDLINE (PubMED), Cochrane Library, CINAHL and Web of Science (WOS) databases from inception to 31st December 2022 using the terms “*cancer*”, “*head and neck neoplasms*”, “*exercise*”, “*rehabilitation*”, “*complications*”, “*muscle contraction*”, “*muscle stretching exercises*” combining with booleans “AND”/“OR”. PEDro scale, Cochrane Risk of Bias Tool and GRADE were used to assess methodological quality, risk of bias and grade of recommendation of included studies respectively. 18 studies (n = 1322) were finally included which 1039 (78.6%) were men and 283 (21.4%) were women. In patients who underwent radio-chemotherapy, overall pain [SMD = − 0.62 [− 4.07, 2.83] CI 95%, Z = 0.35, p = 0.72] and OP [SMD = − 0.07 [− 0.62, 0.48] CI 95%, Z = 0.25, p = 0.81] were slightly reduced with exercise in comparison to controls. Besides, lower limb muscle strength [SMD = − 0.10 [− 1.52, 1.32] CI 95%, Z = 0.14, p = 0.89] and fatigue [SMD = − 0.51 [− 0.97, − 0.057] CI 95%, Z = 2.15, p < 0.01] were also improved in those who receive radio-chemoradiation. In HNC survivors treated with neck dissection surgery, exercise was superior to controls in overall pain [SMD = − 1.04 [− 3.31, 1.23] CI 95%, Z = 0.90, p = 0.37] and, in mid-term, on shoulder pain SMD = − 2.81 [− 7.06, 1.43] CI 95%, Z = 1.76, p = 0.08]. No differences in quality of life were found at any of the follow-up periods. There is evidence of fair to good methodological quality, low to moderate risk of bias, and weak recommendations supporting the use of exercise-based rehabilitation to increase functionality. However, no evidence was found in favor of the use of this modality for improving the quality of life of HNC survivors who underwent chemoradiotherapy or surgery.

## Introduction

Head and neck tumors comprise a heterogeneous group of lesions arising from different structures of the head and neck, with the exception of intracranial, skin, and ocular tumors^1,2^. They present certain characteristics that make them similar, such as being squamous cell carcinomas (HNcSCC) in most cases (95%) and being in easily accessible areas for inspection, which means that they can be detected in early stages^3^. In addition, malignant head and neck cancers (HNC) can histologically present as lymphoepithelioma^4,5^ spindle cell carcinoma^6^, verrucous carcinoma^7^, and undifferentiated carcinoma^8^. HNC accounted for approximately 3% of all cancers in the United States in 2018 and 1.8% of all cancer deaths in the United States during 2020^9^. Particularly, HNcSCC is considered a fatal disease within the first 3.5 years of follow-up, with a relapse mortality rate of 2.3% per year^10^. Depending on anatomical location, they can be located in the lips, pharynx, larynx, paranasal sinus, nasal and oral cavities^11,12^.

These tumors are clearly associated with tobacco and alcohol abuse^13^. Other risk factors include poor oral hygiene, infection of oncogenic viruses as papillomavirus and Epstein-Barr virus and finally Plummer-Vinson syndrome^14–16^.

Treatment for these cancers includes surgery, radiation therapy (RDT), and chemotherapy (CHT)^17,18^. In general, phase I and II outcomes are similar in patients undergone RDT or surgery^19^. In some cases, such as the base of the tongue, stages I and II may require the combination of surgery and RDT, or alternatively, the use of external tele-radiotherapy and brachytherapy^20–22^. Furthermore, advanced stages III and IV are usually treated with a combination of surgery and almost always postoperative RDT^19^. Instead, patients within stages II and IV may be offered the possibility of being treated within clinical trials that purchase CT and RDT and/or radiosensitizers^23,24^.

Rehabilitation with physical activity during and after treatment is an important aspect for cancer survivors, as sequelae are often identified^25–28^. Exercise is known to have positive effects on physical recovery (i.e. *body composition, nutritional status*, etc.), physical function (i.e*. pain, muscle strength, range of motion, fatigue,* etc.), psychological outcomes (i.e*. depression, anxiety,* etc.) and quality of life, such as after breast cancer treatment^29^. Among the many motivations for exercising in the setting of cancer setting include avoiding muscle weakness and atrophy, cardiorespiratory fitness decline and improving energy metabolism efficiency at the cellular level^30–32^. Immunological effects in cancer survivors have also been reported to improve with exercise, including increased cytolytic activity of natural killer cells, the monocyte-functional fraction of circulating granulocytes, and reduced duration of neutropenia^33–35^. Another important reason for cancer patients to exercise is to lose weight, because body mass index is directly proportional to tumor recurrence rate and all-cause mortality^36–38^. Furthermore, obesity has also been described as a risk factor for postoperative lymphedema, assuming loss of function and quality of life^39,40^.

Although it has been studied in other types of cancer, such as breast and prostate cancer, the effectiveness of exercise on disease and treatment related sequelae in HNC have not been thoroughly studied^41,42^. Furthermore, the reviews conducted to date have not been systematic and have provided only partial and, in some cases, circumstantial evidence^43,44^. For all the above reasons, the aim of this meta-analysis was to quantify the effect of exercise-based rehabilitation on functionality and quality of life in HNC survivors who underwent surgery and/or chemoradiotherapy.

## Materials and methods

### Data source and search strategy

A systematic literature review and meta-analysis were carried out regarding the Preferred Reporting Items for Systematic Review and Meta-Analyses (PRISMA)^45^. The protocol for this systematic review was previously registered on PROSPERO (International database for prospectively registered systematic reviews; CRD42023390300).

### Study selection

The selection criteria were: (1) randomized or non-randomized clinical trials, (2) published from the start of the database until December 31, 2022, (3) published in English, Spanish and Portuguese, (4) available in full text, (5) recruiting adults with HNC survivors who have undergone radio-chemotherapy or surgery, and (6) exercise-based program alone or combined with other educational or psychological support that measured (7) functionality and quality of life.

### Search strategy

A literature search was conducted January 10, 2023 to February, 2, 2023 to identify all available studies on the effectiveness exercise-based rehabilitation on functionality and quality of life in HNC survivors in MEDLINE (PubMED), Cochrane Library, CINAHL complete and Web of Science (WOS) databases. In MEDLINE, the search string was “Head and Neck Neoplasms” [Mesh] OR “Exercise” [Mesh] OR “rehabilitation” [Mesh] OR “complications” [Mesh] OR “Muscle Contraction” [Mesh] OR “Muscle Stretching Exercises” [Mesh] OR “cancer” [tw] OR exercis*[tw] OR stretch*[tw] OR plyometric*[tw] OR resist* [tw] OR eccentric [tw] OR concentric [tw] OR isometric*[tw] OR isotonic*[tw] OR activat*[tw] OR contract*[tw] OR conditioning [tw] OR training [tw] *and* “Head and Neck Neoplasms” [Mesh] OR “Exercise” [Mesh] OR “rehabilitation” [Mesh] OR “Muscle Contraction” [Mesh] OR “Muscle Stretching Exercises” [Mesh] OR “lymphedema” [Mesh] OR “quality of life” [Mesh] OR “pain” [Mesh] OR “cancer” [tw] OR function*[tw] OR exercis*[tw] OR stretch*[tw] OR plyometric*[tw] OR resist* [tw] OR eccentric [tw] OR concentric [tw] OR isometric*[tw] OR isotonic*[tw] OR activat*[tw] OR contract*[tw] OR conditioning [tw] OR training [tw]. Similar research equations are used to consult the Cochrane Library, CINAHL and Web of Science (WOS). Two independent researchers (RPG and IMMP) performed the search and a blinded researcher, CRG, scored all retrieved articles by title and abstract, and then scored full-text publications to determine their eligibility. In case of discrepancies, a fourth author served as decision judge (FHR) (Table 1).

**Table 1:** Search strategy.

Search data	Database	Search terms	Search equations
10/01/2023	MEDLINE (PubMED)	“Head and neck neoplasms”, “exercise”, “rehabilitation”, “complications”, “muscle contraction”, “muscle stretching exercises”, “cancer”,“plyometric”, “resistance”, “eccentric”, “concentric”, “isometric”, “isotonic”, “activation”, “contraction”, “conditioning”, “training”	“Head and Neck Neoplasms” [Mesh] OR “Exercise” [Mesh] OR “rehabilitation” [Mesh] OR “complications” [Mesh] OR “Muscle Contraction” [Mesh] OR “Muscle Stretching Exercises” [Mesh] OR “cancer” [tw] OR exercis*[tw] OR stretch*[tw] OR plyometric*[tw] OR resist* [tw] OR eccentric [tw] OR concentric [tw] OR isometric*[tw] OR isotonic*[tw] OR activat*[tw] OR contract*[tw] OR conditioning [tw] OR training [tw]
12/01/2023	MEDLINE (PubMED)	“Head and neck neoplasms”, “exercise”, “rehabilitation”, “muscle contraction”, “muscle stretching exercises”, “lymphedema”, “quality of life”, “pain” “cancer”, “function”, “plyometric”, “resistance”, “eccentric”, “concentric”, “isometric”, “isotonic”, “activation”, “contraction”, “conditioning”, “training”	“Head and Neck Neoplasms” [Mesh] OR “Exercise” [Mesh] OR “rehabilitation” [Mesh] OR “Muscle Contraction” [Mesh] OR “Muscle Stretching Exercises” [Mesh] OR “lymphedema” [Mesh] OR “quality of life” [Mesh] OR “pain” [Mesh] OR “cancer” [tw] OR function*[tw] OR exercis*[tw] OR stretch*[tw] OR plyometric*[tw] OR resist* [tw] OR eccentric [tw] OR concentric [tw] OR isometric*[tw] OR isotonic*[tw] OR activat*[tw] OR contract*[tw] OR conditioning [tw] OR training [tw]
14/01/2023	Cochrane library	“Head and neck neoplasms”, “exercise”, “rehabilitation”, “complication”, “muscle contraction”, “muscle stretching exercises”, “cancer”, “plyometric”, “resistance”, “eccentric”, “concentric”, “isometric”, “isotonic”, “activation”, “contraction”, “conditioning”, “training”	[mh “Head and neck neoplasms”] OR [mh “Exercise”] OR [mh “Rehabilitation”] OR [mh “complications”] OR [mh “Muscle Contraction”] OR [mh “Muscle Stretching Exercises”] OR cancer:ti,ab,kw OR exercis*:ti,ab,kw OR stretch*:ti,ab,kw OR plyometric*:ti,ab,kw OR resist*:ti,ab,kw OR eccentric:ti,ab,kw OR concentric:ti,ab,kw OR isometric*:ti,ab,kw OR isotonic*:ti,ab,kw OR activat*:ti,ab,kw OR contract*:ti,ab,kw OR conditioning:ti,ab,kw OR training:ti,ab,kw)
15/01/2023	Cochrane library	“Head and neck neoplasms”, “exercise”, “rehabilitation”, “muscle contraction”, “muscle stretching exercises”, “lymphedema”, “quality of life”, “pain” “cancer”, “function”, “plyometric”, “resistance”, “eccentric”, “concentric”, “isometric”, “isotonic”, “activation”, “contraction”, “conditioning”, “training”	[mh “Head and neck neoplasms”] OR [mh Exercise] OR [mh “Rehabilitation”] OR [mh “Muscle Contraction”] OR [mh “Muscle Stretching Exercises”] OR [mh “lymphedema”] OR [mh “quality of life”] OR [mh “pain”] OR cancer:ti,ab,kw OR function**:ti,ab,kw OR exercis*:ti,ab,kw OR stretch*:ti,ab,kw OR plyometric*:ti,ab,kw OR resist*:ti,ab,kw OR eccentric:ti,ab,kw OR concentric:ti,ab,kw OR isometric*:ti,ab,kw OR isotonic*:ti,ab,kw OR activat*:ti,ab,kw OR contract*:ti,ab,kw OR conditioning:ti,ab,kw OR training:ti,ab,kw)
21/01/2023	CINAHL	“Head and neck neoplasms”, “exercise”, “rehabilitation”, “complications”, “muscle contraction”, “muscle stretching exercises”, “cancer”, “plyometric”, “resistance”, “eccentric”, “concentric”, “isometric”, “isotonic”, “activation”, “contraction”, “conditioning”, “training”	(“head and neck neoplasm” OR MH “Exercise + ” OR MH “rehabilitation” OR MH “complications” OR MH “Muscle Contraction + ” OR MH “muscle stretching exercises” OR MH “Stretching” OR cancer OR function* exercis* OR stretch* OR plyometric* OR resist* OR eccentric OR concentric OR isometric* OR isotonic* OR activat* OR contract* OR conditioning OR training)
25/01/2023	CINAHL	“Head and neck neoplasms”, “exercise”, “rehabilitation”, “muscle contraction”, “lymphedema”, “quality of life”, “pain” “cancer”, “function”, “stretching”, “plyometric”, “resistance”, “eccentric”, “concentric”, “isometric”, “isotonic”, “activation”, “contraction”, “conditioning”, “training”	(“head and neck neoplasm” OR MH “Exercise + ” OR MH “rehabilitation” OR MH “Muscle Contraction + ” OR MH “lymphedema” OR MH “quality of life” OR MH “pain” OR MH “Stretching” OR cancer OR function* exercis* OR stretch* OR plyometric* OR resist* OR eccentric OR concentric OR isometric* OR isotonic* OR activat* OR contract* OR conditioning OR training)
1/02/2023	Web of Science (WOS)	“Head and neck neoplasms”, “exercise”, “rehabilitation”, “complications”, “post- “muscle contraction”, “muscle stretching exercises”, “cancer”, “plyometric”, “resistance”, “eccentric”, “concentric”, “isometric”, “isotonic”, “activation”, “contraction”, “conditioning”, “training”	“Head and Neck Neoplasms” [Mesh] OR “Exercise” [Mesh] OR “rehabilitation” [Mesh] OR “complications” [Mesh] OR “Muscle Contraction” [Mesh] OR “Muscle Stretching Exercises” [Mesh] OR “cancer” [tw] OR exercis*[tw] OR stretch*[tw] OR plyometric*[tw] OR resist* [tw] OR eccentric [tw] OR concentric [tw] OR isometric*[tw] OR isotonic*[tw] OR activat*[tw] OR contract*[tw] OR conditioning [tw] OR training [tw]
2/02/2023	Web of Science (WOS)	“Head and neck neoplasms”, “exercise”, “rehabilitation”, “muscle contraction”, “muscle stretching exercises”, “lymphedema”, “quality of life”, “pain”, “cancer”, “function”, “plyometric”, “resistance”, “eccentric”, “concentric”, “isometric”, “isotonic”, “activation”, “contraction”, “conditioning”, “training”	“Head and Neck Neoplasms” [Mesh] OR “Exercise” [Mesh] OR “rehabilitation” [Mesh] OR “Muscle Contraction” [Mesh] OR “Muscle Stretching Exercises” [Mesh] OR “lymphedema” [Mesh] OR “quality of life” [Mesh] OR “pain” [Mesh] OR “cancer” [tw] function*[tw] OR exercis*[tw] OR stretch*[tw] OR plyometric*[tw] OR resist* [tw] OR eccentric [tw] OR concentric [tw] OR isometric*[tw] OR isotonic*[tw] OR activat*[tw] OR contract*[tw] OR conditioning [tw] OR training [tw]

### Selection and data extraction

Data extraction was performed independently by two authors (DZL and GBM), and in case of disagreement, a third author (NKY) was the responsible of resolving discrepancies. A standardized work template based on *PICO* question was used to extract and detail all the information related to authors, year and country of publication, study design, aim of the study, outcomes, participants (*characteristic of disease, medical intervention, sample size, gender distribution,* etc.), intervention and control details, results of measured outcomes and conclusions. The Cochrane Handbook for Systematic Reviews of Interventions—v.5.1.0 was used to develop these sections. The reliability of the table was tested using a representative sample of the studies to be reviewed.

### Methodological quality analysis

The PEDro scale were used to assess the methodological quality of clinical trials and were ultimately included in the assessment for this review^46^. It consists of 11 items, each worth one point, and can be used to assess whether a randomized clinical trial has sufficient internal validity (criteria 2–9) and sufficient statistical information to make its results interpretable (criteria 10–9). Studies scoring 9–10 on the PEDro scale were considered to be of excellent methodological quality. Studies with a score between 6 and 8 were of good methodological quality, and studies with a score of less than 4 were of poor methodological quality.

### Risk of bias analysis

Risk of bias analyzes of randomized clinical trials were independently performed by SMP using the Cochrane Risk of Bias Tool for Randomized Trials (RoB 2.0)^47^. The tool evaluates the methods researchers use in clinical trial design and individually rates the presence of the following biases: (1) *randomization process,* (2) *deviations from the intended interventions,* (3) *missing outcome data,* (4) *measurement of the outcome* and (5) *selection of the reported result.* Interpretation of the scores obtained considers the fact that a low risk of bias means that the bias committed is unlikely to significantly alter the results, whereas a high risk of bias indicates lower confidence in the results receive. Any disagreement of the authors was resolved by discussion, and in case of conflicting scores, the third reviewer (FRH) resolved to make the decision.

### Grade of recommendation (GRADE)

The certainty of the evidence analysis was established by the different levels of evidence according to the Grading of Recommendations, Assessment, Development, and Evaluation (*GRADE*) framework which is based on five domains: *study design, imprecision, indirectness, inconsistency,* and *publication bias*^48^. The evidence was classified into the following four levels: high quality (*all domains satisfied*), moderate (*one domain not satisfied*), low quality (*two domains not satisfied*), or very low quality (*three or more domains not satisfied*).

### Data synthesis

Meta-analyses were undertaken using Review Manager (RevMan v.5.3; Cochrane Collaboration, Oxford, UK) when more than two studies reported on the same outcome. In the pooled analysis of studies by duration, outcome data were organized into short-term (≤ 6 weeks), medium-term (7–23 weeks) and long-term (≥ 24 weeks) according to previous studies^49^. In cases where it is not possible to convert the units of measurement, the standardized mean difference is used. Data are presented as standard mean differences (SMD) and 95% CIs. The I^2^ statistic is used to quantify statistical heterogeneity as follows: 0–40%, probably not important; 30–60%, moderate heterogeneity; 50–90%, significant heterogeneity; 75–100%, significant heterogeneity. Analysis was performed using a fixed-effects model, however, when statistical heterogeneity (I^2^ > 40%) was found, a random-effects model was used for meta-analysis.

## Results

### Study selection

**Figure 1:**
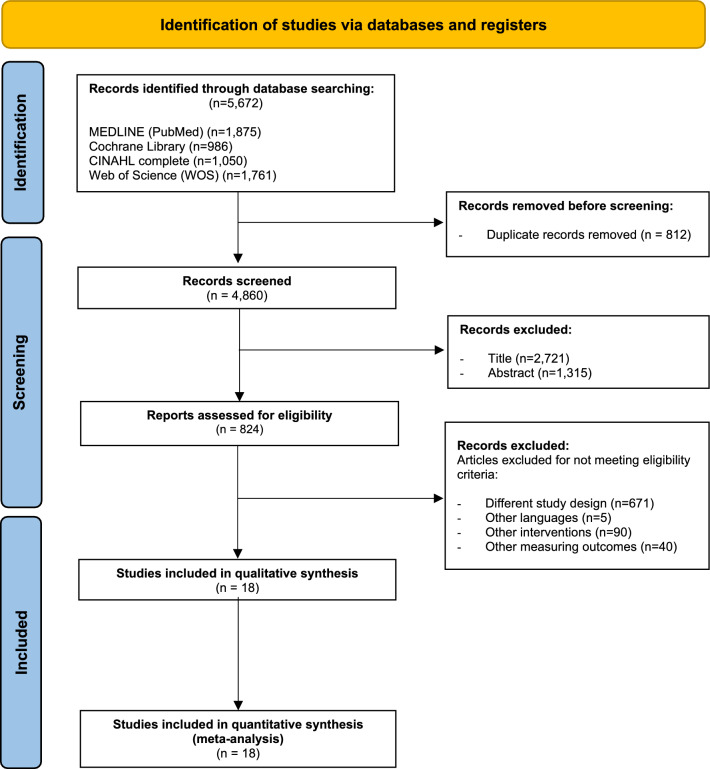
*PRISMA* flow diagram (PRISMA 2020).

A total of 5,672 studies were detected and analyzed by performing the agreed searches in the detailed databases MEDLINE (*PubMed*) (n = 1875), Cochrane Library (n = 986), CINAHL (n = 1050) and Web of Science (*WOS*) (n = 1761). After removing duplicates (n = 812) and analyzing the titles and abstracts of the remaining articles (n = 4036), 824 full-text articles were potentially relevant studies according to the search strategies. Finally, 806 of these manuscripts were excluded because the studies did not meet our eligibility criteria as they had a different study design (n = 671), published in different languages (n = 5), carried out another intervention (n = 90) and have measured other outcomes of little interest for our research question (n = 40). Therefore, 18 studies were ultimately selected for this review for qualitative synthesis and meta-analysis (Fig. 1).

### Study characteristics

The 18 studies were clinical trials which 10 were prospective, parallel, single-blinded, randomized, controlled trials, 3 pilot-controlled trials, 1 randomized, controlled, double-blind, 3-arm, parallel-group, prevention clinical trial, 1 prospective clinical cohort study, 1 single-blinded multicenter, 1 Phase III randomized trial and 1 uncontrolled pre–posttest design. All the included studies were carried out within a population diagnosed with HNC in *oropharynx, hypopharynx, oral cavity, larynx, or unknown primary (UPT)* undergone to RDT, CHT, brachytherapy or modified radical neck dissection reaching a total sample of 1322 patients (n = 1039 men (78.6%) and n = 283 women (21.4%) with a mean age of 53.2. Most of the studies included exercise interventions based on supervised multimodal exercise programs. For example, most studies included aerobic (AET)^50–53^, anaerobic resistance (RET)^50–59^ or endurance^60,61^, stretching^52,57,59,62–64^, relaxation^65^ and postural control exercises^59^ and the goal of increasing joint range in shoulder^59,61,63^ or in cervical spine^57^ in their interventions to improve functionality and quality of life. Most of these developed a protocol of exercise in combination with biomedical education^52,55^, psycho-behavioral couching^30^, diet carried out during CHT^50,56,62,66^ or RDT^53,54,58,62^ alone or combinate radio-chemotherapy^51,67^ or surgery^52,57,59,60,63,65^ with follow-up ranging from 2 weeks to 12 months. The 18 studies included were conducted in Sweden^64,67^, Denmark^54,58^, The Netherlands^60,66^, United States^53,56^, Canada^55,61^, Brazil^62^, India^51,63^, Taiwan^50,52,65^ and Australia^57,59^ (Table 2).

**Table 2:** Study characteristics.

Author, year	Study design	Participants	Intervention	Comparation	Outcomes	Conclusion
Dotevall el al. (2022)^67^ (Sweden)	RCT(*prospective, parallel, single-blinded, randomized, controlled trial*)	n = 47 (M = 25; F = 12) *Inclusion criteria* (1) HNC (*tonsil, base of tongue, hypopharynx, or larynx*) (2) 6 months up to 36 months prior (3) Beam radiation therapy (EBRT) ± brachytherapy or Chemo-radiotherapy	**Nº participants** = 23 **Protocol duration**: 8 weeks **Frequency**: 3 times/daily per week **IG**: Isometric and isokinetic head lifts in supine position Sustained/static head lifts for 60 s three times with 1-min rest between the lifts (isometric training 30 consecutive repetitions of head lifts (isokinetic training)	**Nº participants** = 24 **Protocol duration**: 8 weeks **CG**: Speech language therapy and therapeutic coaching Advice about food, drinking, head position, or swallowing maneuvers, such as the supraglottic swallow, effortful swallow, and the Mendelsohn maneuver during meals	**Follow-up 2 months** **Penetration Aspiration Scale (PAS)** IG = 3.8 (2.0); CG = 4.2 (2.1) Difference between group 95% CI − 0.3 (− 1.3; 0.7); p = 0.62 **Secretion volume** *Thin 3 ml* IG = 2.0 (1.4); CG = 1.9 (1.0) Difference between group 95% CI 0.1 (− 1.0; 1.2); p = 0.91 *Thick 5 ml* IG = 1.7 (1.1);CG = 1.6 (0.9) Difference between group 95% CI − 0.3 (− 0.8; 0.2); p = 0.63 *Thin 10 ml* IG = 2.7 (1.8); CG = 3.0 (1.6) Difference between group 95% CI − 0.1(− 1.4; 1.3); p = 0.58 *Thin 20 ml* IG = 3.4 (1.9); CG = 3.9 (2.3) Difference between group 95% CI − 0.1 (− 1.7; − 1.5); p = 0.61 *Biscuit* IG = 2.3 (2.1); CG = 2.0 (1.0) Difference between group 95% CI − 0.6 (− 1.5; 0.3); p = 0.52 EAT-10 IG = 10.1 (8.1); CG = 12.5 (9.9) Difference between group 95% CI 2.6 (− 0.7; 6);p = 0.98	This randomized study regarding the effect of the HLE demonstrated that swallowing outcome measures used in assessment of FEES did not improve in patients treated with radiotherapy for patients with dysphagia following HNC
Loh et al. (2022)^65^ (Taiwan)	RCT (*prospective, parallel, single-blinded, randomized, controlled trial*)	*n* = *68 (M* = *60;F* = *8)* ***Inclusion criteria*** (1) HNC (2) Age ≥ 20 years (3) no history of surgery for HNC (4) plan to undergo surgery (5) willingness to participate in this study and provide informed consent (6) physically capable of participation the study, as determined by the physician in charge (7) ability to communicate in Mandarin or Taiwanese	**Nº participants** = 34 **Protocol duration**: 10 days post-surgery **Frequency:** 3 h/daily **IG:** Progressive muscle relaxation starting with a comfortable position, closing their eyes, and following the MP3 file instruction	**Nº participants** = 34 **Protocol duration:** 10 days post-surgery **Frequency:** 3 h/daily **CG:** Routine procedure	**Follow-up 10 days** **Pain (VAS)** IG = 0.9 (0.7); CG = 8.7 (4.9); p < 0.01 **Fatigue (VAS)** IG = 55.25 (2.35); CG = 48.64(3.18); p < 0.01 **Sleep (VAS)** IG = 56.89 (3.75); CG = 44.91 (4.68); p < 0.01 **Depression (VAS)** IG = 9.3 (1.68); CG = 6.81 (2.42); p < 0.01 **Anxiety (VAS)** IG = 24.61 (2.0); CG = 12.98 (2.79); p < 0.01 **Respiratory rate (VAS)** IG = 19.7 (0.25); CG = 0.36 (0.27); p < 0.01	PMR can reduce sleep disturbances and levels of pain, fatigue, muscle tightness, anxiety, and depression in patients with head and neck cancer undergoing major surgeries
Hajdú et al. (2022)^54^ (Denmark)	RCT (*prospective, parallel, single-blinded, randomized, controlled trial*)	n = 235 (M = 191; F = 44) ***Inclusion criteria*** *(1)* HNC (*oropharynx, hypopharynx, oral cavity, larynx or unknown primary (UPT)* (2) ≥ 18 years of age (3) eligible for curatively intended RDT treatment	**Nº participants = **120 **Protocol duration:** 8 weeks (follow-ups (6 months and 12 months) **Frequency:** 7 days per week during radiotherapy **Reps:** 10 repetitions **IG:** Exercise programs consisted of all or some of the following 14 exercises: (1) reaching tongue back and (2) forth; (3) tongue to cheek, (4) tongue to mouth corners, (5) resistance to tongue, gargle, (6) yawn, (7) mouth opening, (8) jaw side-to-side, (9) jaw undershot, (10) Valsalva, (11) Shaker exercise, (12) Mendelsohn maneuver, (13) Masako maneuver, (14) Effortful swallow. The PRT program involved 6 exercises covering lower limbs, upper body and core in a fixed progression model based on repetition maximum	**Nº participants** = 115 **CG1**: Not intervention (non-active control group) **CG2**: Tailored exercise plan at beginning of radiotherapy with regular OT follow-up averaging to every other week until 2 weeks after end-of-treatment. (active control group)	**End of treatment** **Mouth opening** Difference between group 95% CI 3.31 (0.86; 5.75); p = 0.01 **Pain (NPRS)** Difference between group 95% CI − 0.96 (− 1.82; − 0.11); p = 0.03 **EORTC QLQ C-30** **Physical functioning** Difference between group 95% CI 7.32 (1.12; 13.52); p = 0.02 **Social functioning** Difference between group 95% CI 12.01 (3.41; 20.52); p = 0.01 **Fatigue** Difference between group 95% CI − 11.15 (− 20.30; − 1.94); p = 0.02 **Insomnia** Difference between group 95% CI − 12.86 (− 24.27; 1.45); p = 0.03 **Appetite loss** Difference between group 95% CI − 17.72 (− 29.11; − 5.93); p = 0.00 **Constipation** Difference between group 95% CI − 16.21 (− 25.87; − 6.55); p = 0.00 **Follow-up 2 months** **EORTC QLQ C-30** ***Role functioning*** Difference between group 95% CI − 14.11 (− 24.36; 3.86); p = 0.01 **Follow-up 6 months** **Insomnia** Difference between group 95% CI − 11.81 (− 23.29; − 0.34); p = 0.04 **Follow-up 12 months** **Mouth opening** Difference between group 95% CI 2.91 (0.43; 5.39); p = 0.02 **MDADI Functional** Difference between group 95% CI − 10.44 (− 19.25; − 1,62); p = 0.02 **Depression** Difference between group 95% CI − 3.69 (− 7.26; − 0.11); p = 0.04 **Anxiety** Difference between group 95% CI − 0.20 (− 0.39; − 0.01); p = 0.04 **Pain** Difference between group 95% CI − 1.25 (− 2.12; 0.38); p = 0.01 **Insomnia** Difference between group 95% CI − 14.07 (− 25.81; − 2.33); p = 0.02	This randomized controlled trial on preventive swallowing exercises and progressive resistance training during RDT did not show an effect on swallowing safety in HNC patients measured by penetration and aspiration However, comparing intervention to a non-active control group only, significant effects were found on mouth opening, HRQOL, depression and anxiety 1 year after end of treatment
Lin et al. (2021)^50^ (Taiwan)	RCT *(prospective, parallel, single-blinded, randomized, controlled trial)*	n = 40 (M = 25; F = 15) ***Inclusion criteria*** (1) adults (≥ 20 years old) (2) a diagnosis of HNC (3) scheduled to receive chemotherapy (4) no brain tumor metastasis (5) no serious complications (6) no history of mental illness	**Nº participants** = 20 **Protocol duration**: 8 weeks **Frequency**: 3 times/daily per week **IG:** Moderate-intensity aerobic, resistance and flexibility exercises supervised by a physical therapist **Duration:** 90 min and included 5-min warm-up and a 5-min cool-down exercises	**Nº participants** = 20 **Protocol duration:** 8 weeks **CG:** Usual care. No specific information regarding exercise and were given general education including information about the side effects of CHT	**Follow-up 2 months** **Body composition** **Body fat percentage** IG = 21.0 (2.8); CG = 25.8 (2.5); p = 0.002 **Skeletal muscle percentage** IG = 34.5 (2.4); CG = 31.4 (2.4); p = 0.008 **Physical Fitness** *Dynamic balance* IG = 6.42 (1.51); CG = 8.4 (1.29); p = 0.01 **Strength (reps/30 s)** *Upper extremity* IG = 27.0 (5.8); CG = 21.06 (5.38); p = 0.037 *Lower extremity* IG = 20.14 (7.04); CG = 13.13 (3.87); p = 0.025	This study found that a combined aerobic, resistance and flexibility exercise program during chemotherapy may improve physical fitness (i.e., muscle strength, balance, flexibility, and body composition) and HRQoL and alleviate the deterioration of cardiovascular fitness in patients with HNC
Bragante et al. (2020)^62^ (Brazil)	RCT (*randomized, controlled, double-blind, 3-arm, parallel-group, prevention clinical trial*)	n = 90 (M = *76*; F = *14*) **Inclusion criteria** (1) HNC (2) age ≥ 18 years (3) Treatment with definitive or postoperative external beam RDT (4) either alone or in combination with chemotherapy or target therapy; and Karnofsky Performance Status (KPS) greater than 60%	**Nº participants** = 30 **Protocol duration:** 7-week beam radiotherapy **Frequency:** 4 times/daily **IG1:** (1) Warm-up (exercises to enhance joint lubrication): 10 times mouth opening and closing, 10 times right lateral excursion, 10 times left lateral excursion, and 10 times mandibular protrusion (2) Stretching: 6 sets of holding the TheraBite device at MMO for 30 s, resting 30 s between each set (3) Masticatory training: 5 min of alternating bilateral chewing with the hyperboloid device **Nº participants** = 30 **IG2:** Same warm-up and masticatory training protocols as in G1, but without the TheraBite stretching step	**Nº participants** = 30 **Protocol duration**: 7-week beam radiotherapy **Frequency:** 4 times/daily **CG:** Not exposed to either of the tested intervention Protocols. controls received the usual care guidance from the nursing team	**Follow-up 7 weeks** **Presence of mucositis** IG = 20.0 (71.4); IG2 = 14 (53.8); CG = 11 (37.9); p < 0.001 **Pain** IG1 = 0.7 (0.4); IG2 = 1.0 (0.4); CG = 1.8 (0.5); p = 0.024 **Follow-up 12 months** **Presence of mucositis** IG = 0 (0.0); IG2 = 1 (4.2); CG = 0 (0.0); p < 0.001	It was not possible to conclude that the exercise protocols performed in this study are more effective than the usual guidance to prevent reduction in MO in patients undergoing RDT for HNC
Thomas et al. (2020)^63^ (India)	RCT *(prospective, parallel, single-blinded, randomized, controlled trial)*	n = *44* (M = *37*; F = *9*) **Inclusion criteria** *(1)* Head and Neck Cancers (2) 30–65 years of age (3) Treatment with MRND	**Nº participants** = 21 **Protocol duration:** 10 days post-intervention **Reps:** 2 sets of 10 repetitions IG: (1) Active range of motion exercises (AROM): Pendulum exercises in standing position, Active assisted ROM in supine position (which was then progressed to active exercises), cross-body adduction in supine position and wall climbing or finger ladder exercises in standing position Shoulder Arm up held at that position for 30 s	**Nº participants** = 25 **Protocol duration:** 10 days post-intervention **Reps:** 2 sets of 10 repetitions ***CG:*** MET exercises which included post isometric relaxation techniques for shoulder flexion, abduction, and internal rotation. Patient position was supine lying. They were asked to contract using 20% of their muscle force. The contraction was held for 7–10 s	**Follow-up 10 days** **Pain (NPRS)** IG = 4.33 ± 1.19; CG = 4.20 ± 1.08 p > 0.05 **AROM Shoulder Abduction** IG = 112.0 (19.98); CG = 110.72 (12.46); p = 0.026 **AROM Shoulder External rotation** IG = 57.9 (9.56); CG = 63.84 (7.87); p = 0.08 **Global Rating Change (GRC)** IG = 1.38 (1.28); CG = − 3.36 (0.64); p = 0.000	METs and AROM exercises were effective in improving shoulder range of motion, function and reducing pain in patients post MRND but-Muscle Energy Techniques were more effective when compared to AROM exercises
Samuel et al. (2019)^51^ (India)	RCT *(prospective, parallel, single-blinded, randomized, controlled trial)*	n = 148 (M = 131; F = 17) **Inclusion criteria** (1) adults (≥ 20 years old) (2) a diagnosis of HNC (3) Treated with radical chemo-radiotherapy	**Nº participants** = 58 **Protocol duration:** 11 weeks (7 weeks of exercise training in the hospital and 4 weeks of home-based exercise program) **Frequency:** 5 times/daily per week IG: (1) Aerobic (brisk walking, 15–20 min) (2) Active resistance exercise program for the major muscles of upper limb and lower limb done in 2 sets (1 set = 8 to 15 repetitions) of *biceps curl, triceps extension, overhead shoulder flexion, hip flexion, quadriceps* (knee extension), and *hip abduction*	**Nº participants** = 62 **Protocol duration:**11 weeks **Frequency:** 5 times/daily per week **CG:** Physical activity recommendations of three 10-min walks during the day for 5 days a week	**Follow-up 3 weeks** **Functional capacity (6MWT)** IG = 437.18 (69.75); CG = 380.74 (105.26); p < 0.001 **Quality of life (SF 36)** *SF36-PCS* IG = 42.21 (6.36); CG = 40.26 (4.74); p < 0.001 *SF36-MCS* IG = 41.26 (8.41); CG = 37.53 (6.64); p < 0.001 **Cancer-related fatigue** IG = 3.81 (1.58); CG = 4.95 (1.33); p < 0.001 **Follow-up 7 weeks** **Functional capacity (6MWT)** IG = 441.72 (90.92); CG = 354.90 (115.66); p < 0.001 **Quality of life (SF 36)** *SF36-PCS* IG = 44.73 (7.15); CG = 37.57 (6.56); p < 0.001 *SF36-MCS* IG = 3.81 (1.58); CG = 4.95 (7.02); p < 0.001 **Cancer-related fatigue** IG = 3.59 (1.78); CG = 5.41 (1.40); p < 0.001 **Follow-up 11 weeks** *Functional capacity (6MWT)* IG = 483.16 (88.24); CG = 374.52 (110.26); p < 0.001 **Quality of life (SF 36)** *SF36-PCS* IG = 48.58 (6.63); CG = 39.10 (4.95); p < 0.001 *SF36-MCS* IG = 43.99 (6.39); CG = 36.34 (5.20); p < 0.001 **Cancer-related fatigue** IG = 2.45 (1.97); CG = 4.48 (1.59); p < 0.001	Our results elucidate that an 11-week structured exercise program for HNC patients receiving CRT helps in improving their functional capacity and QoL. It also prevents deterioration of fatigue levels in the exercise group
Valkenet et al. (2018)^60^ (The Netherlands)	RCT (*single-blind multicentre)*	n = 241 (M = 186; F = 55) **Inclusion criteria** (1) Oesophageal cancer scheduled for transhiatal, transthoracic or minimally invasive (robot-assisted or conventional) oesophagectomy with gastric tube reconstruction	**Nº participants** = 120 **Protocol duration**: 2-weeks pre-surgery **Frequency:** 30 breaths /daily **Duration:** 7 days/week **IG1:** Inspiratory muscle training (1) Load was aimed (60% of maximum inspiratory pressure)	**Nº participants** = 121 **Protocol duration:** 2-weeks pre-surgery **CG:** Usual care	**Baseline** **Respiratory muscle function** **Maximum inspiratory pressure Pi-max (cmH2O)** IG = 76.1 (28.6); CG = 73.0 (30.1); p < 0.001 **Inspiratory muscle endurance Pi-end, m/sec** IG = 4.04 (1.49); CG = 4.17(2.05); p < 0.001 **Lung function measurements** *FEV1 (L, %pred)* IG = 2.8 (93.8); CG = 2.9(95.4) *FVC (L, %pred)* IG = 3.7 (97.0); CG = 3.9(99.2) *FEV1/FVC (%)* IG = 76.3 (11.4); CG = 75.7(10.01) **EQ-5D-3L, quality of life** IG = 0.85 (0.16); CG = 0.87 (0.17) **EQ-5D-3L, VAS health** IG = 74.0 (16.3); CG = 76.1(14.3) *SF-12, Physical Component Scale* IG = 49.2 (9.4); CG = 47. 7 (8.4) *SF-12, Mental Component Scale* IG = 49.2 (9.4); CG = 47.7 (8.4) *MFI-20, physical fatigue score* IG = 10.6 (4.2); CG = 10.2 (4.2) *SQUASH, activity score* IG = 4190 (3533); CG = 3757(3060) **Follow-up 9 days** **Maximum inspiratory pressure Pi-max (cmH2O)** IG = 51.9 (24.8); CG = 45.9 (21.8); p = 0.000 **Lung function measurements** *FEV1 (L, %pred)* IG = 1.6 (53.3); CG = 1.8 (59.0) *FVC (L, %pred)* IG = 2.1 (56.5); CG = 2.4 (62.0) *FEV1/FVC (%)* IG = 74.5 (15.5); CG = 76.1 (12.1) **Follow-up 4 weeks** **EQ-5D-3L, quality of life** IG = 0.73 (0.21); CG = 0.77 (0.18); p = 0.005 **EQ-5D-3L, VAS health** IG = 63.0 (18.8); CG = 66.0 (15.2); p < 0.001 **SF-12, Physical Component Scale** IG = 34.8 (8.5); CG = 34.3 (8.7); p < 0.001 **SF-12, Mental Component Scale** IG = 45.7 (10.5); CG = 48.1 (9.2); p = 0.677 **MFI-20, physical fatigue score** IG = 12.9 (4.1); CG = 13.1 (4.3); p < 0.001 **SQUASH, activity score** IG = 2061 (1952); CG = 1771 (1409); p < 0.001	Standard prescription of IMT before oesophagectomy is not advisable, and IMT programmes aiming to reach high training intensities should probably include supervised elements
Su et al. (2017)^52^ (Taiwan)	RCT (*prospective, parallel, single-blinded, randomized, controlled trial*)	n = 37 (M = 34; F = 3) **Inclusion criteria** (1) primary head and neck malignancy diagnosed within 6 months (2) an age between 20 and 80 years (3) receiving surgical intervention including tumor excision plus selective neck lymph node dissection, with accessory nerve preservation (4) a presurgical malignancy stage of II-IV, based on the Union for International Cancer Control TNM classification system	**Nº participants** = 18 **Protocol duration**: 12-weeks **Frequency**: 5 days /week **Duration**: 60 h **IG:** Home based exercise programme (1) Aerobics (10-min ambulation sessions per week- RPE of 12–13 on the Borg’s RPE 6–20 scale) (2) Anaerobics (middle trapezius, lower trapezius, rhomboid major, biceps brachii, triceps brachii, deltoid, and pectoralis major muscles) **Doses:** 2 sets with 10 repetitions (3) Stretching (Static stretching focused on sternocleidomastoid, upper trapezius, anterior scalene, deltoid, and shoulder internal rotator muscles) **Doses**: 2 times/day 10 s in sets of 5 repetitions with a 15-s rest (4) Education session	**Nº participants** = 19 **Protocol duration:** 12-weeks **Frequency:** 5 days /week **Duration:** 60 h **CG:** Outpatient physiotherapy programme (1) Aerobics (10-min ambulation sessions per week- RPE of 12–13 on the Borg’s RPE 6–20 scale) **Doses:** 30 min (2) Anaerobics (middle trapezius, lower trapezius, rhomboid major, biceps brachii, triceps brachii, deltoid, and pectoralis major muscles) **Doses:** 30% of 1 RM 2 sets 10 repetitions gradually increased by 5% of 1 RM every week, up to 60% of 1 RM as tolerated (3) Stretching (Static stretching focused on exercising the participants’ sternocleidomastoid, upper trapezius, anterior scalene, deltoid, and shoulder internal rotator muscles) Doses: 2 times/day 10 s in sets of 5 repetitions with a 15-s rest (4) Education session	**Follow-up 6 weeks** **Quality of life (FACT H & N)** **IG** = 95.21 (22.27); CG = 94.89 (22.44); p = 0.074 *Shoulder Pain (VAS)* **IG** = 1.11 (1.88); CG = 2.22 (2.60); p = 0.677 **6-min walk test (6MWT)** **IG** = 510.37 (76.58); CG = 508.11 (68.92); p = 0.001 **Shoulder ROM (0°–360°)** Flexion **IG** = 141.28 (17.97); CG = 136.95 (68.92); p < 0.001 Abduction IG = 136.92 (33.74); CG = 141.00 (33.34); p < 0.001 **Follow-up 12 weeks** **Quality of life (FACT H & N)** **IG** = 103.42 (21.48); CG = 93.61 (2.21); p = 0.074 **Shoulder Pain (VAS)** **IG** = 0.58 (1.17); CG = 1.78 (2.69); p = 0.677 **6-min walk test (6MWT)** IG = 556.92 (75.71); CG = 530.47 (75.41); p = 0.001 **Shoulder ROM (0°–360º)** *Flexion* **IG** = 144.44 (18.02); CG = 148.09 (17.51); p < 0.001 *Abduction* **IG** = 140.80 (33.57); CG = 81.82 (10.64); p < 0.001	Both the HBP and OPT can improve shoulder abduction, shoulder flexion and functional capacity
Capozzi et al. (2016)^55^ (Canada)	RCT *(prospective, parallel, single-blinded, randomized, controlled trial)*	n = 60 (M = 49; F = 11) **Inclusion criteria** (1) HNC (2) aged > 18 years (3) newly diagnosed with nasopharyngeal, oropharyngeal, or hypopharyngeal cancer (4) scheduled to receive radiation or concurrent chemoradiation treatment (5) able to walk without assistance (6) received clearance for exercise from their treating oncologist (7) lived in the Calgary area (8) could speak and write English	**Nº participants** = 31 **Protocol duration**: 12 weeks **Frequency**: 2 times/ per week **Duration**: 3 sets of 8 repetitions at 8 RM **IG**: Immediate lifestyle intervention (1) Progressive resistance training **Doses**: Low to moderate intensity Warm-up (5–7 min) 2 sets of 8 repetitions at 8 to 10 repetition maximum (RM) 10 exercises targeting major muscle groups (2) Health education **Doses**: 6 sessions (3) Behavior change support an individualized exercise program a group exercise environment for take advantage of social support **Doses**: Progressive resistance training program with a short, moderate-intensity warm-up (5–7 min) followed by 2 sets of 8 repetitions at 8 to 10 repetition maximum (RM) for 10 exercises targeting major muscle groups	**Nº participants** = 29 **Protocol duration**: 12 weeks **Frequency**: 2 times/ per week **Duration**: 3 sets of 8 repetitions at 8 RM **CG:** Delayed lifestyle intervention (1) Physician referral and clinical support (2) Health education **Doses**: 6 sessions (3) Behavior change support an individualized exercise program a group exercise environment for take advantage of social support **Doses**: Progressive resistance training program with a short, moderate-intensity warm-up (5–7 min) followed by 2 sets of 8 repetitions at 8 to 10 repetition maximum (RM) for 10 exercises targeting major muscle groups	**Follow-up 12 weeks** *Change from Baseline* **Body composition** *Body mass* index (kg/m2) IG = Δ% − 2.5 (0.3); CG = Δ% − 2.8 (0.3); p = 0.387 *Lean body mass (Kg)* IG = Δ% − 4.9 (0.7); CG = Δ% 5.4 (0.7); p = 0.730 *Percentage of body fat* IG = Δ% − 1.5 (0.5); CG = Δ% − 1.9 (0.5); p = 0.658 **Fitness** *Total grip (Kg)* IG = Δ% − 3.0 (1.4); CG = Δ% − 6.7 (1.2); p = 0.734 *6-MWT (meters)* IG = Δ% − 13.0 (19.6); CG = Δ% − 35.4 (18.7); p = 0.590 *Sit to stand (n. of stands)* IG = Δ% 0.6 (0.6); CG = Δ% − 1.9 (0.5); p = 0.536 *Sit-and-reach test (cm)* IG = Δ% − 1.5 (0.5); CG = Δ% − 0.4 (0.5); p = 0.661 **Quality of Life** *FACT, FACT-An* IG = Δ% − 21.3 (4.4); CG = Δ% − 19.1 (4.1); p = 0.751 *FHNSI-22* IG = Δ% − 15.8 (3.0); CG = Δ% − 12.5 (2.8); p = 0.451 *Depression (CES-D)* IG = Δ% 5.1 (1.9); CG = Δ% 4.9 (1.7); p = 0.865 *Nutrition status (PG-SGA)* IG = Δ% 4.4 (1.5); CG = Δ% 6.7 (1.4); p = 0.365 **Follow-up 24 weeks** *Change from Baseline* **Body composition** *Body mass* index (kg/m2) IG = Δ% − 3.2 (0.5); CG = Δ% − 3.3 (0.5); p = 0.310 *Lean body mass (Kg)* IG = Δ% − 4.5 (1.1); CG = Δ% 4.4 (1.0); p = 0.578 *Percentage of body fat* IG = Δ% − 3.5 (0.8); CG = Δ% − 4.3 (0.7); p = 0.796 **Fitness** *Total grip (Kg)* IG = Δ% 0.2 (2.0); CG = Δ% − 1.3 (1.8); p = 0.881 *6-MWT (meters)* IG = Δ% 43.5 (26.3); CG = Δ% 17.8 (24.8); p = 0.665 *Sit to stand (n. of stands)* IG = Δ% 1.7 (0.8); CG = Δ% 2.5 (0.7); p = 0.386 *Sit-and-reach test (cm)* IG = Δ% 2.6 (3.8); CG = Δ% 3.2 (1.1); p = 0.716 **Quality of Life** *FACT, FACT-An* IG = Δ% − 1.0 (5.7); CG = Δ% − 3.0 (5.3); p = 0.765 *FHNSI-22* IG = Δ% − 2.6 (3.8); CG = Δ% − 2.1 (3.5); p = 0.982 *Depression (CES-D)* IG = Δ% − 0.2 (2.4); CG = Δ% 2.7 (2.2); p = 0342 *Nutrition status (PG-SGA)* IG = Δ% 0.6 (1.7); CG = Δ% 0.5 (1.6); p = 0.846	Although the intervention during treatment did not reduce the loss of lean body mass, delaying the exercise program until after treatment completion was associated with improved intervention adherence, a finding with important clinical implications
Cnossen et al. (2016) (The Netherlands)	RCT (*prospective clinical cohort study*)	n = 50 (M = 39; F = 11) **Inclusion criteria** (1) age > 18 years (2) cancer originating in the oral cavity, oropharynx, hypopharynx, or larynx, (3) SW-IMRT alone or in combination with CHT [(C)SW-IMRT] (4) performance status 0–2 on the World Health Organization Scale (5) the absence of severe cognitive impairment (6) sufficient mastery of the Dutch language (criteria 4–6 as judged by the radiation oncologist who included the patients in this study)	**Protocol duration:** 12 weeks **Frequency:** 15 min/day **IG:** Swallowing sparing intensity modulated radiation therapy (SW-IMRT) (1) exercises to maintain mobility of the head, neck, and shoulders (2) exercises to optimize and maintain swallowing function (3) exercises to optimize and maintain vocal health and vocal function (4) exercises to optimize and maintain speech function and functional communication Follow-up: Contacted by phone in a weekly 10-min coaching session by an experienced speech therapist	**CG:** Uncontrolled	**Follow-up 6 weeks** *Treatment* IG OR = 5 (22); CG OR 18 (78); p = 0.015 **Follow-up 12 weeks** *Treatment* IG OR = 6 (26); CG OR 17 (74); p = < 0.001 **EORTC-QLQ-H&N35** *Mouth opening problems* OR = 0.91 (0.84–0.99) p = 0.037	Adherence to a guided home-based prophylactic exercise program was high during (C)SW-IMRT, but dropped afterwards. Exercise performance level was negatively affected by chemotherapy in combination with SW-IMRT
Zhao et al. (2016)^56^ (United States)	RCT (*Pilot controlled trial*)	n = 18 **Inclusion criteria** (1) 40 years or older (2) with American Joint Committee on Cancer stage II to IV head and neck squamous cell carcinoma (3) who were beginning first-line concurrent CRT without surgery (4) who were capable of understanding and adhering to the protocol requirements	**Nº participants** = 11 **IG1: MPACT intervention** Protocol duration: 14 weeks (1) warm-up (rhythmic large muscle movements, such as marching and punching, whereas the cool down included leg, shoulder, and arm flexibility activities coupled with deep breathing) (2) Strengthening, cardiovascular fitness, and physical activity components (3) Cool-down (4) Rest **IG2: Functional resistance training** Protocol duration: 7 weeks Reps: 8 to 12 repetitions (1) Chest press in squat (2) wall push up (3) military press (4) side arm raises (5) biceps curl (6) shoulder shrugs (7) calf raises Weights included dumbbells and inserts into an ankle strap **IG3: Walking** Protocol duration: 7 weeks Frequency: 5 min. 6 times/day Total walking time of 30 min IG4: Home program Frequency: 5 days a week and a minimum of 30 min per day, performed in bouts of 10 min or more, at a moderate intensity Functional resistance in addition to walking and physical activity program was customized based on: (1) personal determinants (self-efficacy, benefits, and barriers) (2) physical activity preferences (3) available community resources (4) health and environmental factors	**Nº participants** = 7 **Protocol duration:** 14 weeks **CG:** Controls received standard treatment, including active nutritional surveillance, but were neither encouraged nor discouraged to exercise	**Follow-up 7 weeks** *Physical performance* **Knee extension strength, N-m** IG = 1.0 (11.00); CG = − 36.0(16.0); p < 0.05 *SF-36 subscale: vitality* IG = − 19.0 (7.00); CG = − 33.0 (3.0); p < 0.05 **SF-36 subscale: mental health** IG = 3.0 (4.0); CG = − 16.0(7.0); p < 0.05 **Follow-up 14 weeks** *Mean (SE) change* **Physical performance** *Knee extension strength, N-m* IG = − 4 (7.0); CG = − 46.0 (14.0); p < 0.05	In this pilot study of patients with HNC undergoing concurrent CRT, MPACT training was feasible and maintained or improved function and QoL, thereby providing the basis for larger future interventions with longer follow-up
McGarvey et al. (2015)^57^ (Australia)	RCT *(prospective, parallel, single-blinded, randomized, controlled trial)*	n = 59 (M = 43; F = 16) **Inclusion criteria** (1) carcinoma of the head and neck region and had undergone a neck dissection, with accessory nerve preservation (2) within the past 8 weeks before study entry, with demonstrated clinical signs of accessory nerve shoulder dysfunction after surgery (3) fully healed neck dissection scar (4) ≥ 18 years of age (5) and ability to sufficiently communicate in the English language	**Nº participants** = 32 **Protocol duration**: 12 weeks **Frequency**: 1 supervised session and 2 home sessions per week **Doses**: 2 to 3 sets **Reps**: 8–12 **Rest**: 1-min rest **IG:** (1) Progressive scapular strengthening exercises of the upper trapezius, rhomboid, and serratus anterior muscles, utilizing hand weights, with the lowest possible weight being 0.5 kg (2) Active cervical spine range of motion exercises in all directions (10–15 repetitions, 1–2 sets), active-assisted shoulder range of motion exercises (10–15 repetitions, 1–2 sets), cervical spine and pectoralis major stretches (30-s hold, 3 repetitions) (3) Advice of self-administered scar tissue massage	**Nº participants** = 27 **Protocol duration**: 12 weeks **CG:** (1) General advice and a (2) Brochure of generalized shoulder and neck exercises (*photographs of active-assisted glenohumeral joint exercises, active cervical spine range of movement exercises, and advice about scar tissue massage, correct posture, and encouraging functional use of the upper limb*)	**Follow-up 3 months** **Shoulder Pain and Disability Index (SPADI)** Difference between group 95% CI 4.34 (− 9.10, 17.77) **AROM Shoulder Abduction** Difference between group 95% CI 11.92 (− 7.4, 31.2) **AROM Shoulder Flexion** Difference between group 95% CI − 2.65 (− 16.1, 10.8) **Neck Dissection Impairment Index (NDII)** Difference between group 95% CI − 6.16 (− 18.5, 6.2) **Follow-up 6 months** **Shoulder Pain and Disability Index (SPADI)** Difference between group 95% CI 2.97 (− 11.3, 17.2) **AROM Shoulder Abduction** Difference between group 95% CI − 3.0 (− 23.5, 17.5) **AROM Shoulder Flexion** Difference between group 95% CI − 6.0 (− 20.3, 8.3) **Neck Dissection Impairment Index (NDII)** Difference between group 95% CI − 5.26 (− 18.3, 7.8) **Follow-up 12 months** **Shoulder Pain and Disability Index (SPADI)** Difference between group 95% CI − 5.26 (− 9.8, 20.4) **AROM Shoulder Abduction** Difference between group 95% CI − 5.56 (− 27.5, 16.4) **AROM Shoulder Flexion** Difference between group 95% CI − 6.39 (− 21.6, 9.0) **Neck Dissection Impairment Index (NDII)** Difference between group 95% CI − 8.25(− 22.1, 5.6)	The intervention is a favorable treatment for maximizing shoulder abduction in the short term. The effect of the intervention com- pared to usual care is uncertain in the longer term
Pauli et al. (2015)^64^ (Sweden)	RCT *(prospective, parallel, single-blinded, randomized, controlled trial)*	n = 50 (M = 31; F = 19) **Inclusion criteria** (1) Head and Neck Cancer (2) Trismus-related symptoms	**Nº participants** = 25 **Protocol duration**: 10 weeks **Frequency:** 5 times /day **IG:** TheraBite (1) warm-up movements consisting of jaw opening 10 times and small sideway movements of the jaws 10 times without using the jaw device (2) Passive stretching, with the jaw mobilizing device, 30 s repeated 5 times (3) 5 repetitions of active exercise (bite toward resistance) (4) Passive stretching	**Nº participants** = 25 **Protocol duration:** 10 weeks **Frequency**: 5 times /day **IG:** Engström jaw mobilizing device (1) warm-up movements consisting of jaw opening 10 times and small sideway movements of the jaws 10 times without using the jaw device (2) passive stretching, with the jaw mobilizing device, 30 s repeated 5 times (3) 5 repetitions of active exercise (bite toward resistance) (4) Passive stretching	**Follow-up 3 months** **Maximal interincisal opening (MIO)** IG = 39.9(37.9–41.9); CG = 37.4(34.2–40.4); p = 0.256 **Trismus-related** **Symptoms (Gothenburg Trismus Questionnaire)** ***Jaw-related problems*** IG = 20.2 (14.3, 26.0); CG = 25.7 (15.6–35.7); p < 0.001 ***Eating limitation*** IG = 30.0 (20.6–39.4); CG = 26.3 (16.0–36.5); p < 0.05 **Muscular tension** IG = 10.7 (6.1–15.3); CG = 15.7 (9.7–21.7); p < 0.05 **Facial pain** ***Facial pain right now*** IG = 8.7 (1.2–16.1); CG = 9.3 (3.7–15.0); p < 0.001 ***Facial pain when worst last mo*** IG = 24.0 (14.3–33.7); CG = 21.3 (12.6–30.1); p < 0.001 ***Facial pain average value last mo*** IG = 21.3 (12.8–29.9); CG = 20.7 (12.2–29.2); p < 0.001 ***Facial pain affecting ability to work last mo*** IG = 14.0 (2.8–25.2); CG = 13.0 (1.8–24.2); p < 0.01 **LOM** IG = 34.0 (23.3–44.7); CG = 32.0 (21.9–42.1); p < 0.01 **LOM interfering with social, leisure,** **and family activities last mo** IG = 14.0 (4.1–23.9); CG = 19.0 (5.3–32.7); p < 0.01	Jaw exercise therapy effectively improved mouth opening capacity and led to less trismus-related symptoms. Both jaw devices were proved efficient and compliance to exercise was comparable
Lønbro et al. (2013)^58^ (Denmark)	RCT (*Phase III randomised trial*)	n = 41 (M = 23; F = 16) **Inclusion criteria** (1) Histologically diagnosed with squamous cell carcinoma of the larynx (except glottic stage I + II), pharynx, oral cavity or in lymph nodes from an unknown primary tumor (stage I–IV, TNM, 2002) (2) no current or previous malignancies, psychological, social, or geographical conditions that could prevent participation and training (3) no excessive alcohol intake (4) WHO performance status 0–1 (5) age > 18 years (6) completed curative radio- therapy ± chemotherapy (7) complete tumor regression after treatment (8) written consent	**Nº participants** = 20 **Protocol duration**: 12 weeks **Frequency:** 2–3 sets **Reps:** 8–15 repetitions **IG:** Progressive resistance training- Early Exercise (EE) (1) Leg press (2) Knee extension (3) Hamstring curls (4) Chest press (5) Sit ups (6) Back extensions (7) Lateral pull down	**Nº participants** = 21 **Protocol duration**: 12 weeks **CG:** Progressive resistance training- Delayed Exercise (EE) Limitless self-chosen physical activity	**Follow-up 12 weeks** **Lean body mass (kg)** IG = 54.5 ± 11.0; CG = 55.8 ± 7.8; p = 0.005 **Isometric KE (Nm)** IG = 202 ± 79; CG = 177 ± 48; p = 0.025 **Isokinetic KF (Nm)** IG = 97 ± 33; CG = 95 ± 26; p = 0.005 **30 s chair rise (reps)** IG = 23 ± 5; CG = 21 ± 6; p < 0.05 **Global Health** IG = 74 ± 20; CG = 78 ± 18; p < 0.05 **Physical Function** IG = 89 ± 11; CG = 87 ± 10; p < 0.001 **Role Function** IG = 82 ± 22 CG = 89 ± 13; p < 0.05 **Fatigue** IG = 30 ± 21; CG = 29 ± 17; p < 0.05 **Follow-up 24 weeks** Physical Function **IG = **7 ± 16; **CG = **14 ± 14**; p < 0.05 Role Function **IG = **17 ± 33**; CG = 19.0** 23 ± 23**p < 0.01 **Emotional Role** **IG = **21 ± 23**; CG = **4 ± 19**; p < 0.05** **Social Function** **IG = **18 ± 15*; CG = 6 ± 21**; p < 0.05** **Fatigue** **IG = 14.0 (4.1–23.9); CG = 19.0 (5.3–32.7); p < 0.01**	PRT effectively increased lean body mass and muscle strength in HNSCC patients following radiotherapy, irrespectively of early or delayed start-up
Rogers et al. (2013)^53^ (United States)	RCT (*Pilot controlled trial*)	n = 15 (M = 12; F = 3) **Inclusion criteria** (1) cancer of the oral cavity, pharynx, larynx, nasal cavity/sinuses, or salivary gland (2) > 18 years of age (3) English speaking (4) radiation therapy planned or underway for < 1 week	**Nº participants** = 7 **Protocol duration**: 12 weeks **Frequency:** 2 times/week supervised sessions during the first 6 weeks. 2 times/week of home-based sessions supported with weekly telephone counseling, written materials, and DVD during the next 6 weeks **Duration**: supervised session was 1 h, with the maximum time being 1 h 15 min **IG**: Up to 10 repetitions of 9 different exercises using each of the major muscle groups: (1) Chest press (2) Leg extension (3) Lateral row (4) Reverse curl (5) Triceps using wall push-up (6) Triceps kickback (7) Heel raise (8) 2-arm front raise (9) Hamstring or arm curl	**Nº participants** = 8 **Protocol duration**: 12 weeks **CG:** No specific recommendations regarding engaging or not engaging in aerobic or resistance exercise was provided to the participants randomized to the control group	**Follow-up 12 weeks** **Back/leg extensor strength, kgs** IG = 115.0 (54.4); CG = 92.1 (41.3); *d* = − 0.19 **Chair rise time, sec** IG = 2.9 (0.7); CG = 3.1 (0.4); *d* = − 0.60 ***Right hand grip, kgs*** IG = 39.3 (9.8); CG = 35.5 (11.6); *d* = 0.34 **Lean body mass, lbs** IG = 113.8 (32.9); CG = 132.4 (32.8); *d* = − 0.40 **Body mass index** IG = 26.4 (9.9); CG = 29.9 (7.2); *d* = − 0.29 **Physical functioning** IG = 10.2 (1.3); CG = 8.8 (2.0); *d* = 0.19 **Fatigue** IG = 19.0 (10.0); CG = 16.5 (11.1); *d* = − 0.27	Resistance exercise is safe and feasible in patients with head and neck cancer receiving radiation; a definitive trial is warranted
Eades et al. (2011) (Canada)	RCT (*uncontrolled pre-post test design*)	n = 27 (M = 22; F = 5) **Inclusion criteria** (1) cancer of the oral cavity, pharynx, larynx, nasal cavity/sinuses, or salivary gland (2) > 18 years of age	**Nº participants** = 27 **Protocol duration:** 8 weeks **Frequency:** 2 times/week **IG**: CNR (Cancer Nutrition-Rehabilitation) program (1) range of motion (2) endurance (3) mobility training (for example, transfers, gait, stair climbing)	**CG**: Uncontrolled	**Follow-up 8 weeks** Symptom severity *Pain* Difference between group 95% CI 1.8 (1.0, 2.6); *d* = 0.9 *Weakness* Difference between group 95% CI 1.89 (0.9, 2.9); *d* = 0.8 *Shortness of breath* Difference between group 95% CI 2.0 (0.8, 3.1); *d* = 0.7 *Anorexia* Difference between group 95% CI 2.0 (0.7, 3.3); *d* = 0.7 *Insomnia* Difference between group 95% CI 1.5 (0.5, 2.5); *d* = 0.6 *Depression* Difference between group 95% CI 1.8 (1.0, 2.6); *d* = 0.9 *Distress* Difference between group 95% CI 1.8 (1.0, 2.6); *d* = 0.9 *Quality of life* Difference between group 95% CI 1.8 (1.0, 2.6); *d* = 0.9	An interdisciplinary rehabilitation program may be beneficial to patients with head and neck cancer after treatment, but its effects should be evaluated in a controlled trial
McNeely et al. (2008) ^59^ (Canada)	RCT (*Pilot controlled trial)*	n = 52 (M = 37; F = 15) **Inclusion criteria** 1) surgical treatment, including radical neck dissection, MRND, and other variants of selective neck dissection 2) Karnofsky performance status > 60% 3) no evidence of residual cancer in the neck and no distant (M0) metastasis 4) completion of adjuvant HNC treatment (4) with symptoms of shoulder dysfunction attributed to spinal accessory nerve damage (5) Shoulder dysfunction because of spinal accessory nerve dysfunction (6) with > 3 months of the following signs: atrophy of the upper trapezius muscle, shoulder droop, scapular malalignment (including lateral drift and rotation of the scapula), winging of the scapula with elevation of the arm (7) limitation in shoulder abduction range of motion (ROM)	**Nº participants** = 27 **Protocol duration**: 12 weeks **Frequency**: 2 sets of 10 to 15 repetitions of 5 to 8 exercises, starting at 25% to 30% of their 1-repetition maximum (1-RM) strength and slowly progressing to 60% to 70% of their 1-RM strength by the end of the intervention period **IG:** Progressive Resistance Exercise Training Group (tailored and supervised) (1) supervised active and passive ROM/stretching exercises (2) postural exercises (3) strengthening exercises with light weights (1–5 kg) and elastic resistance bands **Targets:** rhomboids/middle trapezius; levator scapula/upper trapezius; biceps; and triceps, deltoid, and pectoralis major	**Nº participants** = 25 **Protocol duration:** 12 weeks **CG:** Standardized Therapeutic Exercise Group (1) supervised active and passive ROM/stretching exercises (2) postural exercises (3) basic strengthening exercises with light weights (1–5 kg) and elastic resistance bands **Targets:** rhomboids/middle trapezius; levator scapula/upper trapezius; biceps; and triceps, deltoid, and pectoralis major	**Follow-up 12 weeks** **SPADI Disability subscale** IG = 7.6 (10.1); CG = 16.1 (14.6); p = 0.337 **NDII** IG = 68.6 (22.0); CG = 60.2 (21.9); p = 0.278 **FACT-An (score, 0–188)** IG = 142.4 (27.0); CG = 134.4 (34.0); p = 0.287 **FACT-G (score, 0–108)** IG = 83.9 (15.6); CG = 78.1 (19.3); p = 0.287 **Fatigue subscale (score, 0–52)** IG = 36.7 (9.0); CG = 34.1 (11.1); p = 0.478 **1 RM 2-arm** *Seated row, kg* IG = 60.2 (21.1); CG = 41.3 (23.1); p < 0.001 *Chest press, kg* IG = 51.4 (20.6); CG = 37.0 (21.1); p = 0.007 **1 RM affected shoulder** *Seated row, kg* IG = 27.6 (10.3); CG = 20.6 (11.1); p = 0.003 *Chest press, kg* IG = 24.0 (10.7); CG = 17.5 (9.8); p = 0.001 **Standard load, reps 3 kg** *Endurance test* IG = 1032 (432); CG = 712 (415); p = 0.017	The PRET program significantly reduced shoulder pain and dis- ability and improved upper extremity muscular strength and endurance in head and neck cancer survivors who had shoulder dysfunction because of spinal accessory nerve damage. Clinicians should consider the addition of PRET in the rehabilitation of postsurgical head and neck cancer survivors

AROM: Active range of motion; CES-D: Center for Epidemiologic Studies Depression Scale; CNR: Cancer Nutrition-Rehabilitation; DEXA; Dual Energy X-ray Absorptiometry; EBRT: External Beam radiation therapy; FACT-An: Functional Assessment of Cancer Therapy-Anemia; F*ACT*-*H&N*: Functional Assessment of Cancer Therapy-Head and Neck; FHNSI-22: National Comprehensive Cancer Network‐Functional Assessment of Cancer Therapy (FACT)‐Head and Neck Symptom Index‐22; FACT-An: Functional Assessment of Cancer Therapy-Anemia; FACT-G: Functional Assessment of Cancer Therapy – General; FVC: *forced vital capacity;* ESAS: Symptom severity and quality of life (Edmonton Symptom Assessment System; EQ-5D-3L: EuroQol five-dimensional questionnaire; EORTC-QLQ-H&N35: ORTC questionnaire for the assessment of quality of life in head and neck cancer patients; FEES: flexible endoscopic evaluation of swallowing; FEV1: Forced expiratory volume in the first second; FEV1/FVC (%): Forced expiratory volume/*forced vital capacity ratio;* HBP: Home based programme; HLE: head-lift exercise; HNC: Head and neck cancer; HNSCC: neck squamous cell carcinoma; HRQOL: Health related-Quality of life; KPS: Karnofsky Performance Status; LOM: limitation opening mouth; MFI-20: Multidimensional Fatigue Inventory; MIO: Maximal interincisal opening; MMO: Maximum mouth opening; MET: Muscle Energy Techniques; MRND: Modified Radical Neck Dissection; MO: Mouth opening; MPACT: maintain physical activity during cancer treatment; NDII: Neck Dissection Impairment Index; SF36-PCS: Physical component; SF36-MCS: Mental component; 6MWT: Six minutes walking test; SW-IMRT: swallowing sparing intensity modulated radiation therapy; OPT: Outpatient physiotherapy; OT: Occupational therapy; PG-SGA: Patient-Generated Subjective Global Assessment Short Form; PMR: progressive muscle relaxation; PRET: Progressive resistance exercise training; PRT: Progressive resistance training; RDT: Radiotherapy; RPE: rating of perceived exertion; TNM: T*umor*, Node, *Metastasis*; UPT: unknown primary Tumor.

### Methodological Quality assessment (PEDro Scale)

The methodological quality of the studies included in our review was good (PEDro score = 6.38 out of 10, SD = 1.09). During methodological quality assessment, 14 studies of good quality^50–53,55–60,62,63,65,67^ and 4 studies of acceptable methodological quality were identified^54,61,64,66^. Most of the studies made systematic errors in relation to the blinding of the participants, since of the total that were included, only 1 carried out the blinding of the participants^50^. Also, the blinding of the therapists who applied treatment was only carried out in a study^54^ (Table 3).

**Table 3:** Randomized Clinical trials Methodological quality assessment (*PEDro Scale*).

Author, Year	Score (0–10)	Quality	1	2	3	4	5	6	7	8	9	10	11
Dotevall et al. (2022)^67^	6	Good	Yes	Yes	Yes	Yes	No	No	No	Yes	No	Yes	Yes
Hajdú et al. (2022)^54^	5	Acceptable	Yes	No	No	No	No	Yes	No	Yes	Yes	Yes	Yes
Loh et al. (2022)^65^	7	Good	Yes	Yes	Yes	Yes	No	No	Yes	Yes	Yes	No	Yes
Lin et al. (2021)^50^	7	Good	Yes	Yes	Yes	Yes	Yes	No	No	No	Yes	Yes	Yes
Bragante et al. (2020)^62^	8	Good	Yes	Yes	Yes	Yes	No	No	Yes	Yes	Yes	Yes	Yes
Thomas et al. (2020)^63^	6	Good	Yes	Yes	Yes	Yes	No	No	No	Yes	No	Yes	Yes
Samuel et al. (2019)^51^	6	Good	Yes	Yes	Yes	Yes	No	No	No	Yes	No	Yes	Yes
Valkenet et al. (2018)^60^	8	Good	Yes	Yes	Yes	Yes	No	No	Yes	Yes	Yes	Yes	Yes
Cnossen et al. (2017)^66^	5	Acceptable	Yes	Yes	No	Yes	No	No	No	Yes	No	Yes	Yes
Su et al. (2017)^52^	6	Good	Yes	Yes	No	Yes	No	No	No	Yes	Yes	Yes	Yes
Capozzi et al. (2016)^55^	6	Good	Yes	Yes	No	Yes	No	No	Yes	No	Yes	Yes	Yes
Zhao et al. (2016)^56^	6	Good	No	Yes	No	Yes	No	No	Yes	Yes	No	Yes	Yes
Pauli et al. (2015)^64^	5	Acceptable	Yes	Yes	No	Yes	No	No	No	Yes	No	Yes	Yes
McGarvey et al. (2015)^57^	8	Good	Yes	Yes	Yes	Yes	No	No	Yes	Yes	Yes	Yes	Yes
Lønbro et al. (2013)^58^	6	Good	Yes	Yes	No	Yes	No	No	Yes	Yes	No	Yes	Yes
Rogers et al. (2013)^53^	7	Good	Yes	Yes	Yes	Yes	No	No	No	Yes	Yes	Yes	Yes
Eades et al. (2013)^61^	5	Acceptable	Yes	Yes	No	Yes	No	No	No	Yes	No	Yes	Yes
McNeely et al. (2008)^59^	8	Good	Yes	Yes	Yes	Yes	No	No	Yes	Yes	Yes	Yes	Yes

Result on the PEDro scale: 9–10 (excellent), 6–8 (good), 4–5 (acceptable) and < 4 (poor).

### Risk of bias assessment (ROB 2.0)

The risk of bias of the included randomized clinical trials, as measured by the ROB 2.0 tool, can be marked as low to moderate risk of bias. When analyzing each bias, we found a high risk of bias related to the deviations from intended interventions in most of the included studies^50–53,55–67^, mainly due to lack of blinding of participants and personnel. In relation to the selection of the reported outcomes^51,58,61,63–67^, we detected a high risk of bias since the researchers did not report the outcome in the study variables in detail. With regard to the randomization process, there is a high risk of bias due to the lack of concealment-related errors in the random assignment sequence^52,54,56,58,61,66^ (Table 4).

**Table 4:**
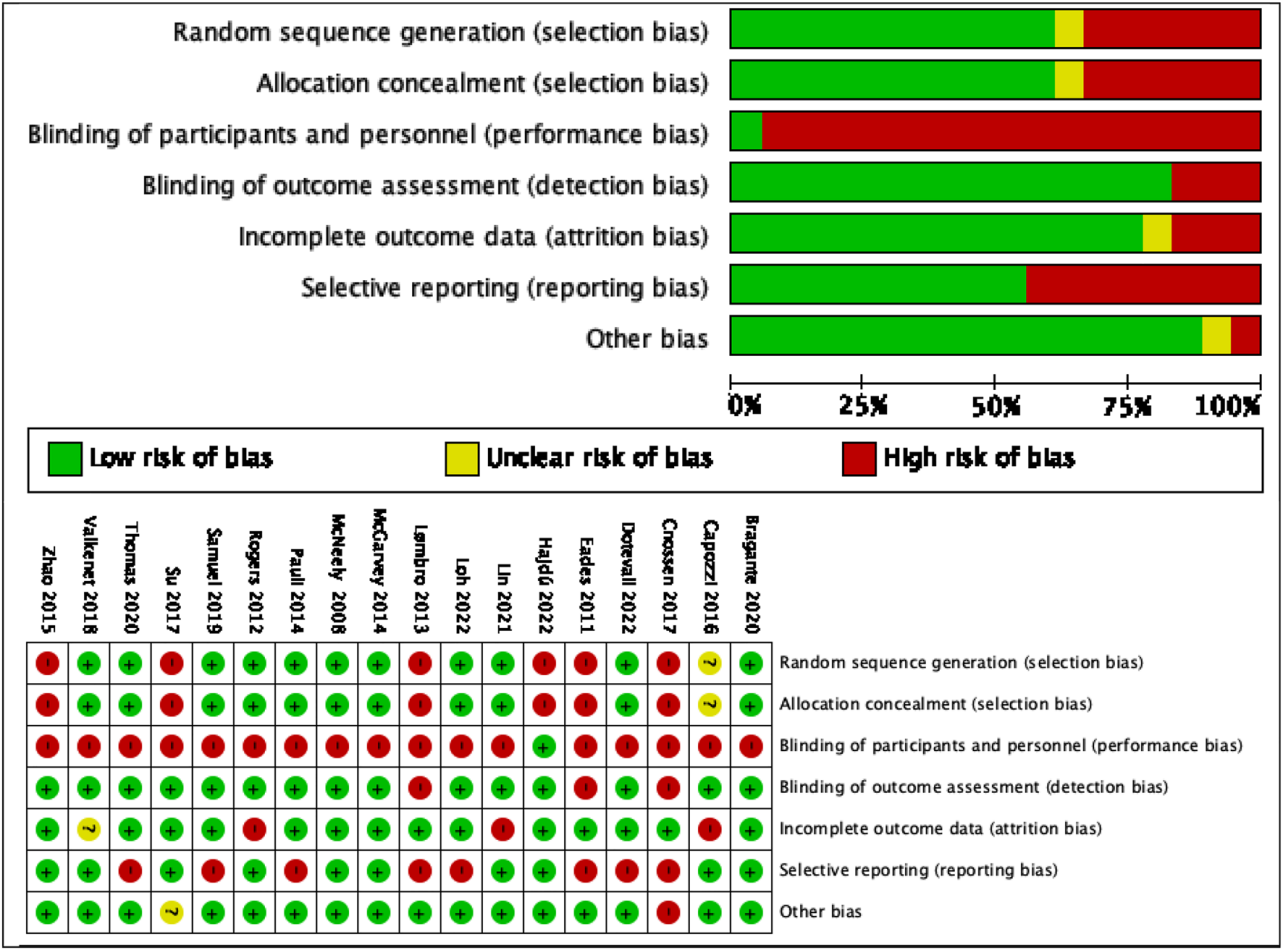
Methodological quality evaluation of the clinical trials using the Cochrane Risk of Bias Tool for assessing the risk of bias in randomized trials (*ROB 2.0*).

Domains: (1) randomization process, (2) deviations from the intended interventions, (3) missing outcome data, (4) measurement of the outcome and (5) selection of the reported result.

### Grade of recommendation (GRADE)

The Grade of recommendation is weak in favor of exercise to improve functionality and quality of life in survivors with HNC (Table 5).

**Table 5:** Summary of findings for clinical trials, including the GRADE quality of evidence assessment.

Number of studies (subjects)	Risk of bias	Inconsistency	Indirectness	Imprecision	Publication bias	Quality	Grade of recommendation
Quality Assessment of Exercise-based rehabilitation Studies on functionality in HNC patients							
17 (n = 1254)	Serious *	Serious^‡^	Not serious	Not serious	Not serious	Low quality	Weak in favor
Quality Assessment of Exercise-based rehabilitation Studies on quality of life in HNC patients							
10 (n = 905)	Serious*	Serious^‡^	Not serious	Not serious	Not serious	Low quality	Weak in favor

*Blinding and/or allocation concealment issues.

^‡^Point estimates varied among studies. The GRADE system establishes 4 degrees of evidence (high, moderate, low, and very low), and 2 degrees of recommendation (strong or weak) for or against the intervention; For each item a judgment is made (very serious, serious, not serious).

### Data synthesis

#### Effectiveness of exercise-based rehabilitation in HNC survivors undergone chemo-radiotherapy

##### Functionality related to pain

**Figure 2:**
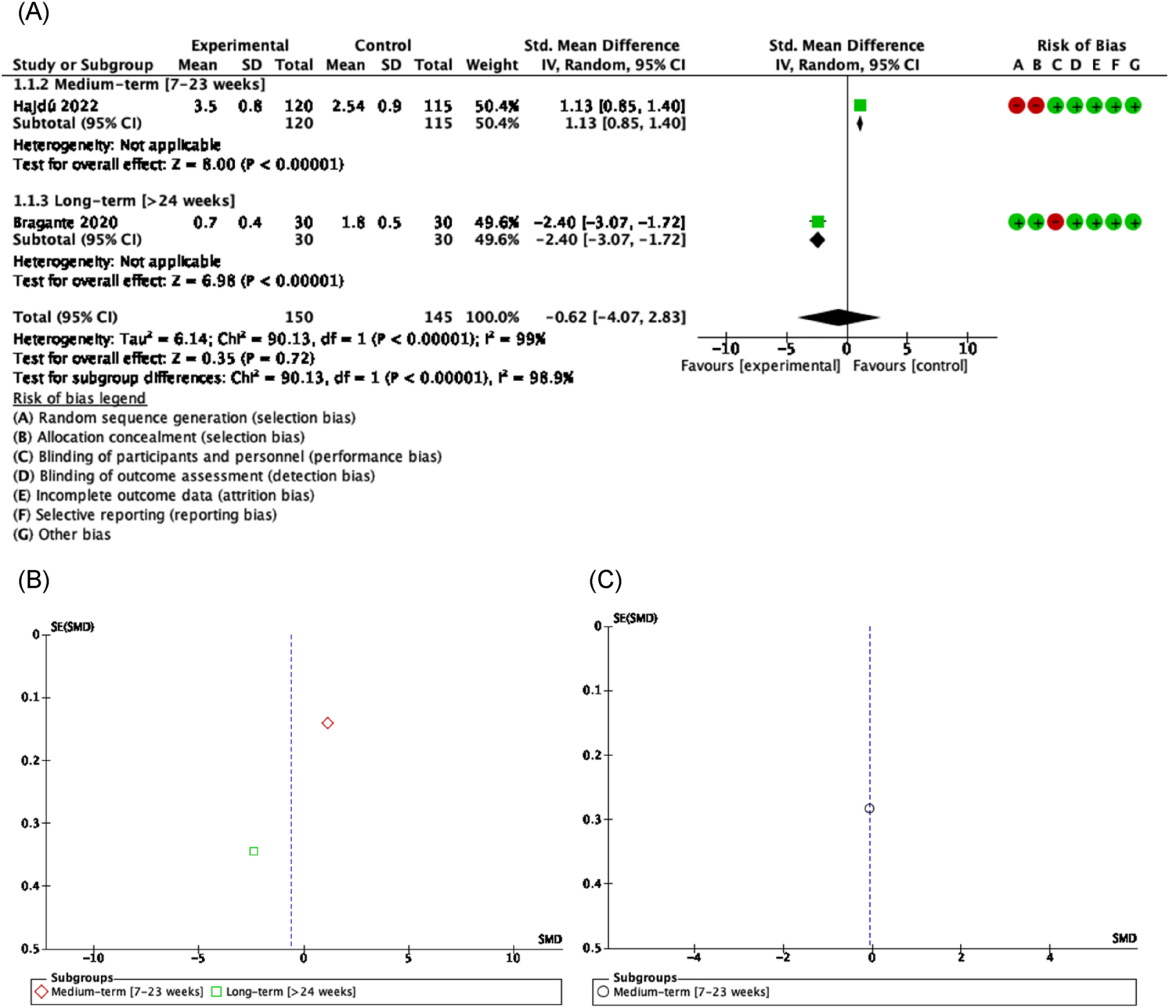
Forest plot and Funnel Plot of the effect of the exercise on functionality related to pain in HNC undergone radio-chemotherapy. (**A**) Forest plot of the effect of the exercise on functionality related to pain in HNC undergone radio-chemotherapy. (**B**) Funnel plot of comparison: Exercise vs Control n HNC survivors undergone Chemo-radiotherapy, outcome: Overall Pain. (**C**) Funnel plot of comparison: Exercise vs Control n HNC survivors undergone Chemo-radiotherapy, outcome: Orofacial Pain.

According to our results, there was evidence of acceptable to good methodological quality, low to moderate risk of bias and significant heterogeneity [Tau^2^ = 6.14; I^2^ = 99%] that showed little size effect of exercise to reduce overall pain in HNC patients underwent chemo-radiotherapy [SMD = − 0.62 [− 4.07, 2.83] CI 95%, Z = 0.35, p = 0.72]^54,62^ (Fig. 2).

##### Functionality related to muscle strength

**Figure 3:**
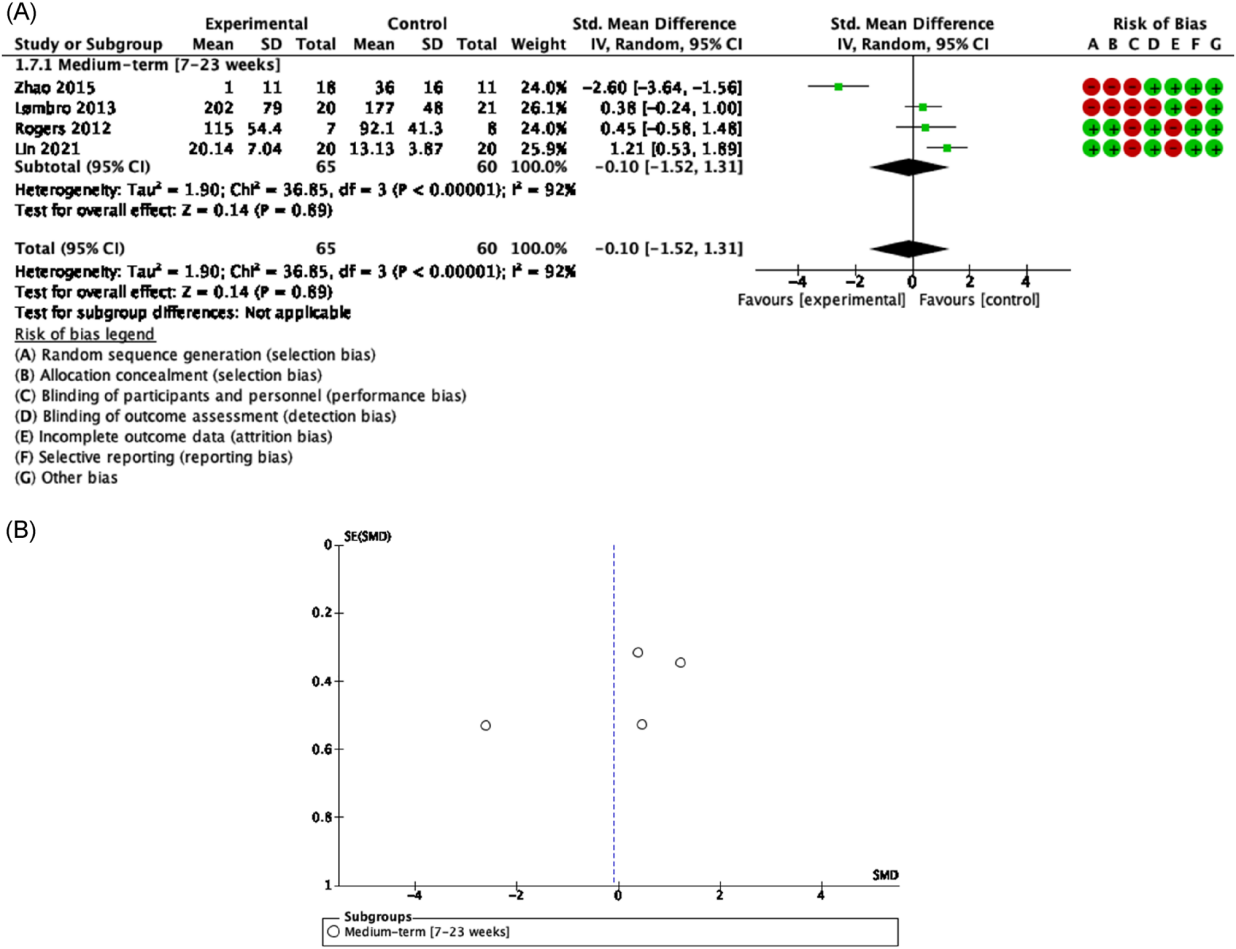
Forest plot and Funnel Plot of the effect of the exercise on functionality related to muscle strength in HNC undergone radio-chemotherapy. (**A**) Forest plot of the effect of the exercise on functionality related to muscle strength in HNC undergone radio-chemotherapy. (**B**) Funnel plot of comparison: Exercise vs Control n HNC survivors undergone Chemo-radiotherapy, outcome: Lower Limb Muscle strength.

There was evidence of good to moderate methodological quality, low to moderate risk of bias, and significantly heterogeneity [Tau^2^ = 0.79; I^2^ = 86%], suggesting no positive treatment effect on upper limb isometric strength in the exercise group compared with controls at medium term [SMD = 0.80 [0.14, 1.46] CI 95%, Z = 0.45, p = 0.66]^50,53^. A little improvement has been reported on long-term [SMD = − 0.57 [− 1.09, − 0.05] CI 95%, Z = 2.16, p = 0.03]^30^ being the overall effect not in favor of exercise [SMD = 0.25 [− 0.484, 1.34] CI 95%, Z = 0.45, p = 0.66] in patients that received radiation or concurrent chemoradiation. In contrast, there were positive results, despite the very small effect size, favoring the use of isometric lower body strength exercises [SMD = − 0.10 [− 1.52, 1.32] CI 95%, Z = 0.14, p = 0.89] in HNC for managing patients scheduled to receive radio-chemotherapy^50,53,56,58^ (Fig. 3).

##### Functionality related to fatigue

**Figure 4:**
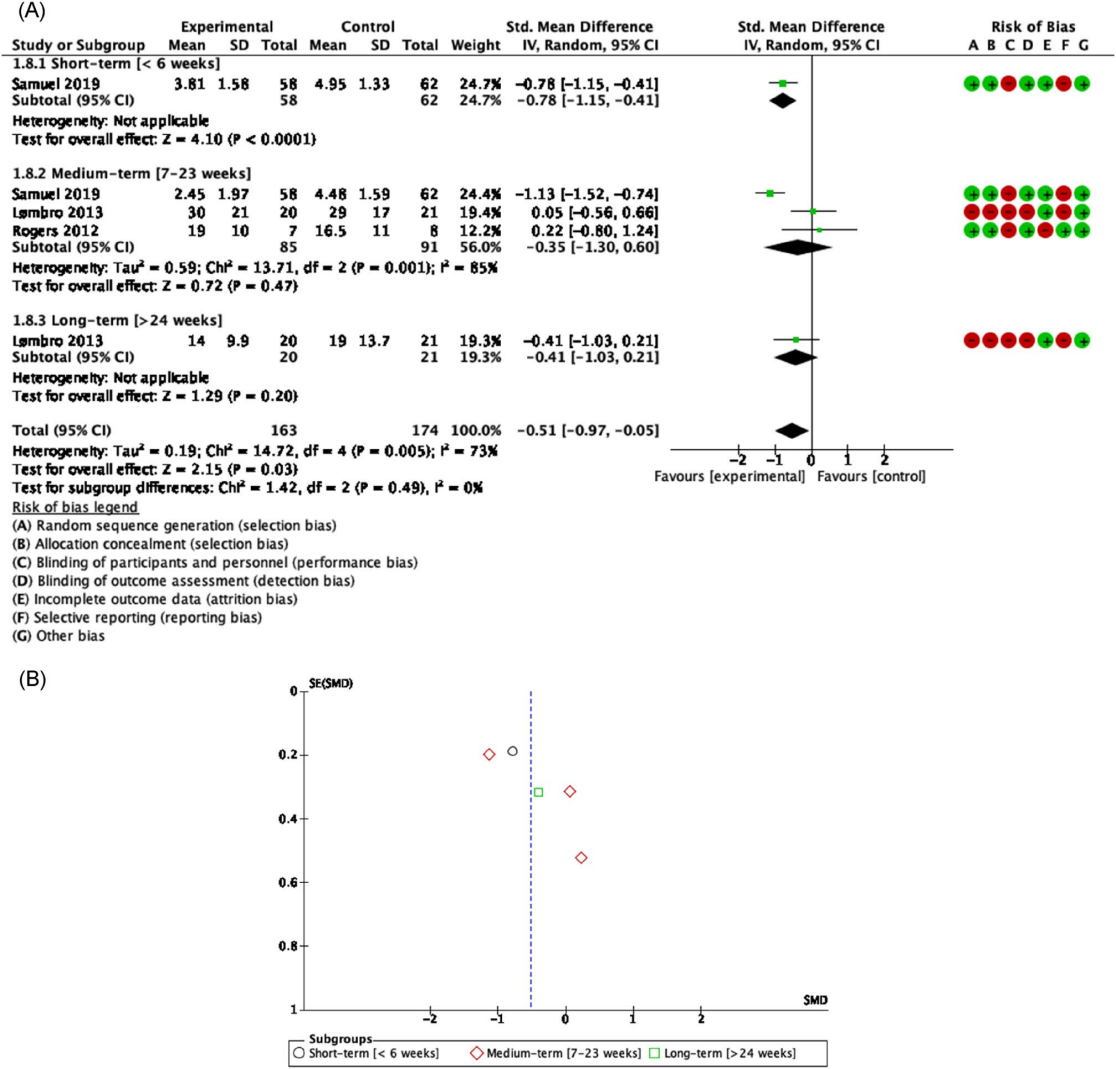
Forest plot and Funnel Plot of the effect of the exercise on functionality related to fatigue in HNC undergone radio-chemotherapy. (**A**) Forest plot of the effect of the exercise on functionality related to fatigue in HNC undergone radio-chemotherapy. (**B**) Funnel plot of comparison: Exercise vs Control n HNC survivors undergone Chemo-radiotherapy, outcome: Fatigue.

There was evidence of good methodological quality, moderate risk of bias, and moderate heterogeneity [Tau^2^ = 0.19; I^2^ = 73%] that showed a significant efficacy of exercise in cancer-related fatigue in HNC who were treated with RDT in all terms of following-up [SMD = − 0.51 [− 0.97, − 0.057] CI 95%, Z = 2.15, p < 0.01]. In the short term, exercise was minimally superior to controls in reducing fatigue perceived by HNC patients who were treated with chemo-radiotherapy [SMD = − 0.78 [− 1.15, − 0.41] CI 95%, Z = 4.10, p < 0.01]^51^. In the medium term, these clinical differences in favor of exercise remain the same [SMD = − 0.35 [− 1.30, 0.60] CI 95% Z = 0.72, p = 0.47]^51,53,58^ as in the long term [SMD = − 0.41 [− 1.03, 0.21] CI 95% Z = 1.29 p = 0.20]^58^ (Fig. 4).

##### Functionality related to range of motion

Evidence of acceptable methodological quality, moderate risk of bias and significantly heterogeneity [Tau^2^ = 0.57; I^2^ = 92%] showed exercise was not superior over controls regarding the range of motion of mouth opening [SMD = 0.65 [− 0.44; 1.74] CI 95%, Z = 1.16, p = 0.24] after performing joint mobility and RET combined with stretching in patients curatively intended RDT treatment^54,64^.

##### Quality of life

**Figure 5:**
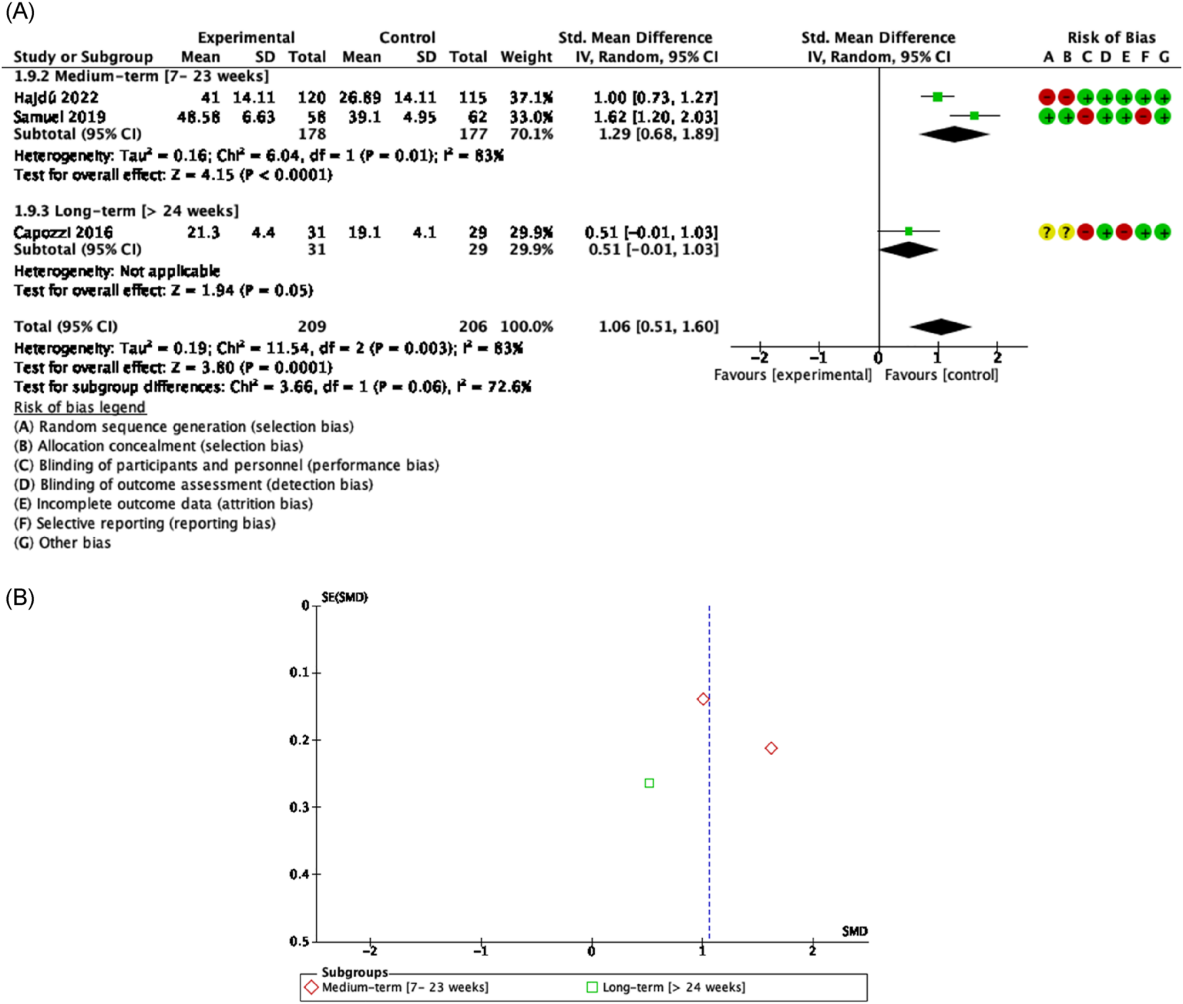
Forest plot and Funnel Plot of the effect of the exercise on functionality related to quality of life in HNC undergone radio-chemotherapy. (**A**) Forest plot of the effect of the exercise on functionality related to quality of life in HNC undergone radio-chemotherapy. (**B**) Funnel plot of comparison: Exercise vs Control n HNC survivors undergone Chemo-radiotherapy, outcome: Quality of life.

There was evidence of acceptable to good methodological quality, moderate risk of bias, and high heterogeneity [Tau^2^ = 0.19; I^2^ = 83%] that showed nor efficacy of exercise in quality of life in comparison to controls [SMD = 1.06 [0.51, 1.60] CI 95% Z = 3.80, p < 0.01]. If we perform an analysis by time, no effects were found in favor of exercise in any of the study terms, short term [SMD = 1.29 [0.68, 1.89] CI 95%, Z = 4.15, p < 0.01]^51,54^ and long term [SMD = 0.51 [− 0.01, 1.03] CI 95%, Z = 1.94, p = 0.05]^55^ in patients with HNC underwent chemoradiation (Fig. 5).

#### Effectiveness of exercise-based rehabilitation in HNC survivors undergone surgery

##### Functionality related to pain

**Figure 6:**
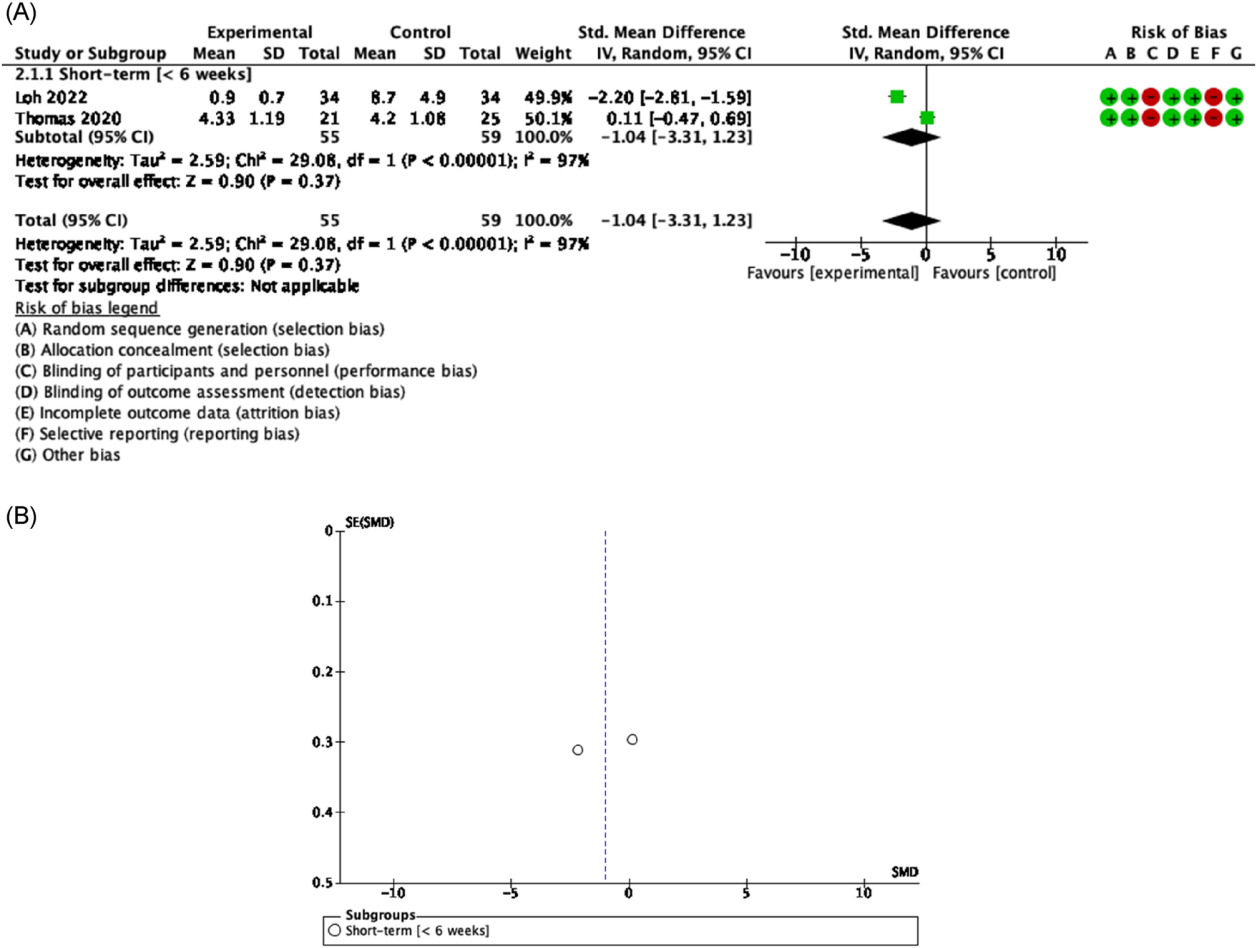
Forest plot and Funnel Plot of the effect of the exercise functionality related to pain (overall pain) in HNC undergone surgery. (**A**) Forest plot of the effect of the exercise on functionality related to pain (overall pain) in HNC undergone surgery. (**B**) Funnel plot of comparison:Exercise vs Control in HNC survivors undergone surgery, outcome: Overall Pain.

**Figure 7:**
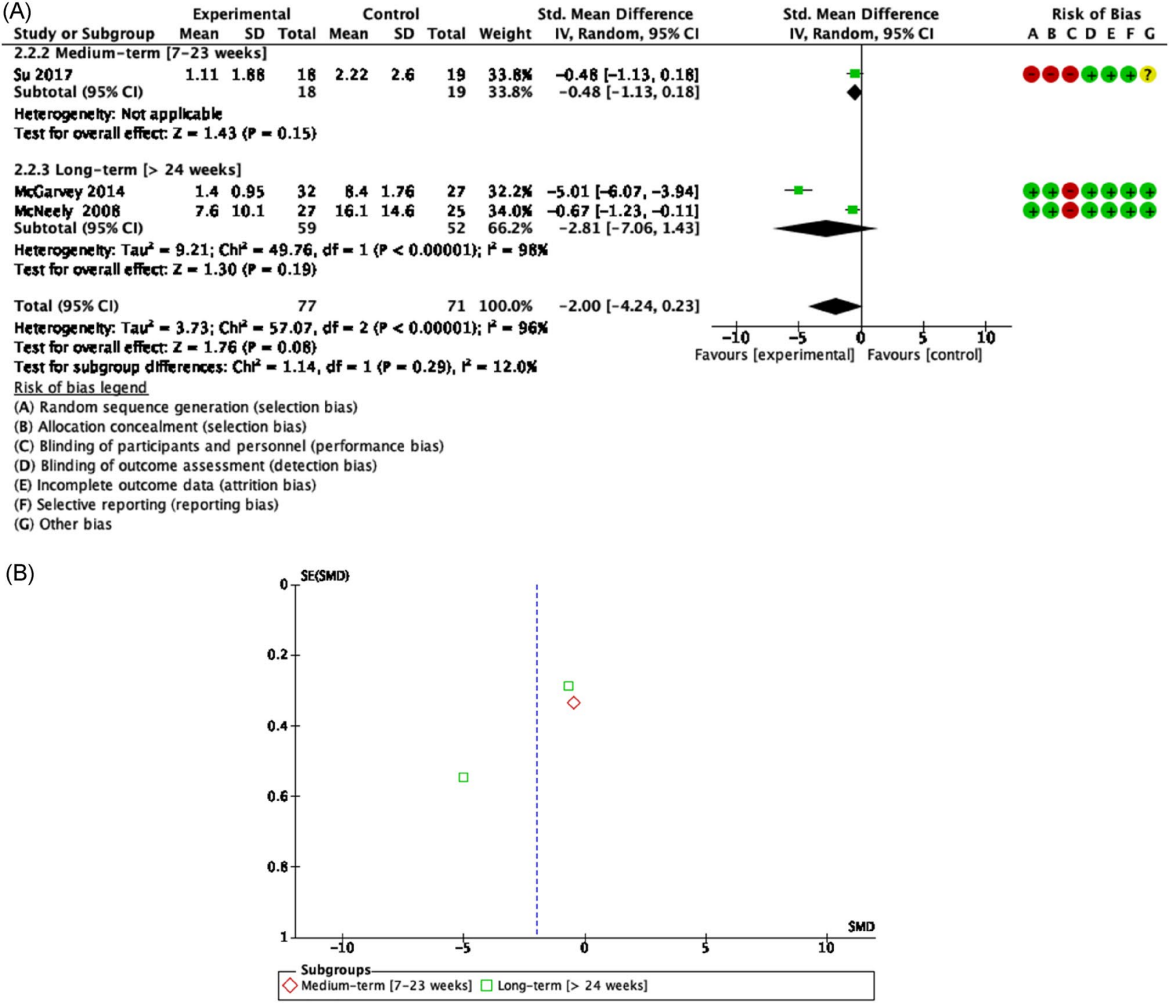
Forest plot and Funnel Plot of the effect of the exercise functionality related to pain (shoulder pain) in HNC undergone surgery. (**A**) Forest plot of the effect of the exercise on functionality related to pain (shoulder pain) in HNC undergone surgery. (**B**) Funnel plot of comparison: Exercise vs Control in HNC survivors undergone surgery, outcome: Shoulder Pain.

Evidence of good methodological quality, moderate risk of bias, and high heterogeneity [Tau^2^ = 2.59; I^2^ = 97%] detected improvements but not statistically significant on overall pain when HNC patients participated in a RET, joint mobility and stretching program after neck dissection surgery [SMD = − 1.04 [− 3.31, 1.23] CI 95%, Z = 0.90, p = 0.37]. Moreover, most effects in favor exercise of RET, joint mobility and relaxation exercise have been showed concerning shoulder pain in the short [SMD = − 0.48 [− 1.13, 0.18] CI 95%, Z = 1.43, p = 0.15]^52^ and long term [SMD = − 2.81 [− 7.06, 1.43] CI 95%, Z = 1.76, p = 0.08] underwent HNC surgery management^57,59^ (Figs. 6, 7).

##### Functionality related to muscle strength

There was a limited evidence of good methodological quality, moderate risk of bias, and high heterogeneity showing exercise based on progressive RET was not superior over controls [SMD = 0.84 [0.27, 1.41] CI 95%, Z = 2.90, p = 0.004] in patients with HNC treated by radical neck dissection^59^.

##### Functionality related to fatigue

**Figure 8:**
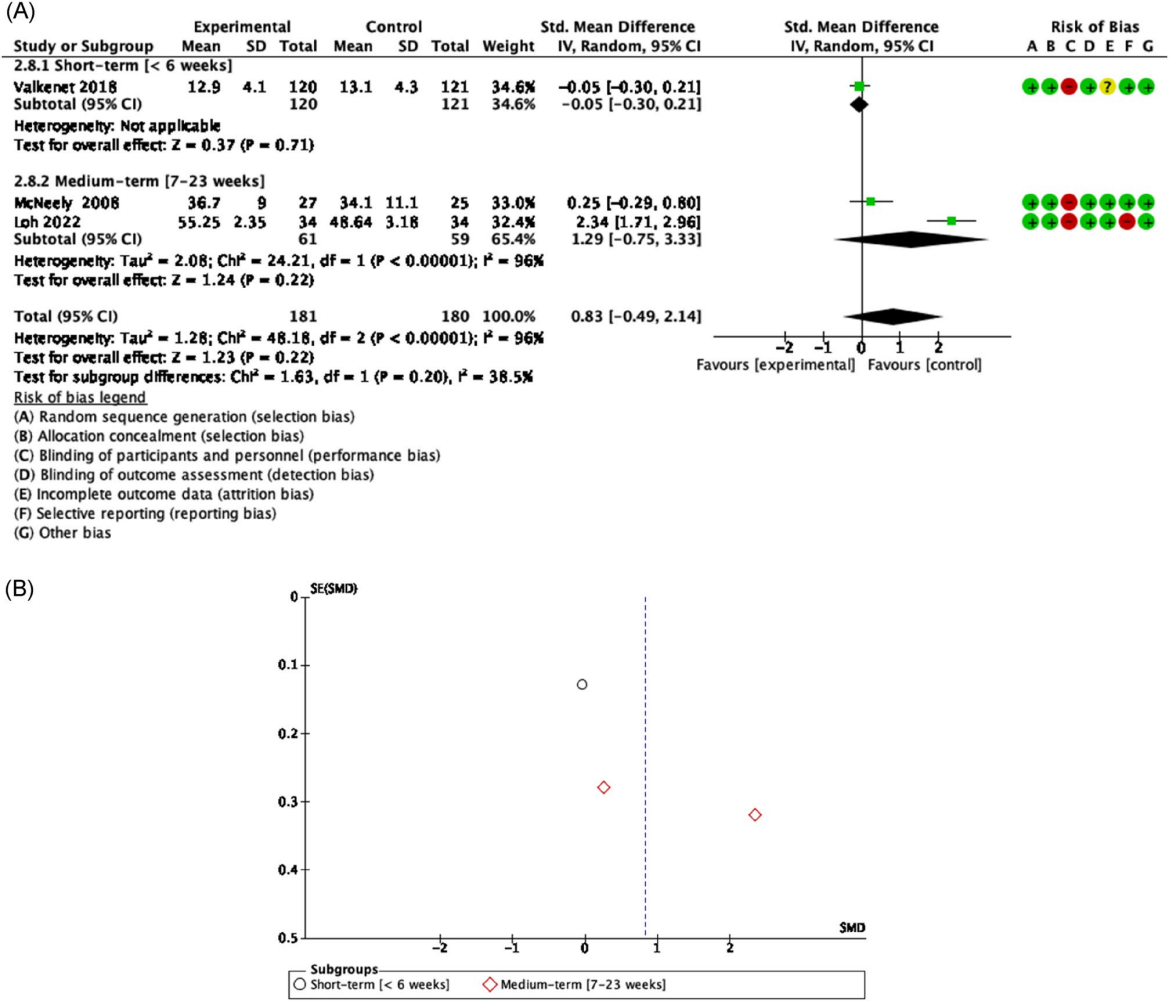
Forest plot and Funnel Plot of the effect of the exercise functionality related to fatigue in HNC undergone surgery. (**A**) Forest plot of the effect of the exercise on functionality related to fatigue in HNC undergone surgery. (**B**) Funnel plot of comparison: Exercise vs Control in HNC survivors undergone surgery, outcome: Fatigue.

Evidence of good methodological quality, moderate risk of bias, and high heterogeneity [Tau^2^ = 1.28; I^2^ = 96%] showed there were not differences of exercise based on progressive RET^59^ or muscle relaxation^65^ versus controls [SMD = 0.83 [− 0.49, 2.14] CI 95%, Z = 1.23, p = 0.22] in patients with HNC treated by radical neck dissection. In contrast, there was only a limited and not lasting of short-term effect on cancer-related fatigue in a group who performed inspiratory muscle training^60^. [SMD = − 0.05 [− 0.30, 0.21] CI 95%, Z = 0.37, p = 0.71] (Fig. 8).

##### Functionality related to range of motion

No differences have been found between exercise and controls in HNC survivors treated by surgery. Evidence of good methodological quality, moderate risk of bias, and high heterogeneity [Tau^2^ = 92; I^2^ = 90%] showed practicing exercise after MRND was not superior to promote active shoulder abduction in any of following-up terms [SMD = 0.89 [− 0.26, 2.04] CI 95%, Z = 1.52, p = 0.13]^52,57,63^.

##### Quality of life

There was evidence of good methodological quality, moderate risk of bias, and probably not important heterogeneity [Tau^2^ = 0.00; I^2^ = 0%] that stablishes there were not better results in relation to quality of life in comparison with controls of HNC surgically treated [SMD = − 0.41 [0.10, 0.73] CI 95%, Z = 2.56, p = 0.01]^52,60^.

## Discussion

### Effectiveness of exercise-based rehabilitation in HNC survivors undergone chemo-radiotherapy

The aim of this meta-analysis was to quantify the effect of exercise-based rehabilitation on functionality and quality of life in HNC survivors who underwent surgery and/or chemoradiotherapy. This modality has a positive effect on overall pain in HNC survivors undergoing chemoradiation. Bragante et al. found that RET combined with joint mobility training and stretching reduce overall pain on long-term in patients undergoing CHT or RDT compared to controls^62^. Cancer pain use to be neuropathic and can be caused both in the Central Nervous System (CNS) and the Peripheral Nervous System (PNS). During tumor growths, compression, and even invasion, can occur triggering painful sensation. CHT and RDT contributes to this neuropathic cancer pain (NCP)^68^. RDT induced pain could be attributed to fibrosis or sensitization of peripheral nerves^69^. Although the underlying effects of the exercise on NCP are not fully understood different hypotheses are postulated. One of the most plausible hypotheses is that dynamic RET produces hypoalgesia by the activation of baroreceptors induced blood pressure changes which are linked to central pain inhibition pathway^70^. Other potential mechanism is that RET increases not only delayed-onset muscle soreness (DOMS), responsible of the increase muscle pain thresholds, but also a stimulation of low-threshold motor units. Both joint mobility and stretching exercises activate centers of descending inhibitory opioid dependent axes and others non-opioids pathways^71^.

Although no positive results were found in favor of exercise in patients with HNC undergoing radio-chemotherapy, Pauli et al.^64^ found that RET combined with joint mobility and stretching exercises reduce OP in the medium term^64^. OP is a localized pain disorder in face and jaw that causes moderate to severe deterioration of chewing^72^. OP uses to be the result of a combination of several joint, myofascial and/or neuropathic mechanisms^73^. In the study of Pauli et al.^64^ pain can be attributed to radio-chemotherapy as well. In this sense RDT, when it acts in the environment of the temporomandibular joint (TMJ), provokes radiation induced fibrosis that arise NCP^69,74^. RET progressively stimulates a local anti-inflammatory response and activation of central microglial activity^75^ that explains the amelioration in neuropathic OP as it is observed in Pauli et al.^64^.

Despite not positive effects of exercise on upper limb muscle strength were observed in patients who received radio-chemotherapy, Capozzi et al. found that the combination of RET with health education and behavioral therapy has a positive effect on the long-term enhancement of upper limb muscles in patients receiving CHT or RDT^30,55^. All these patients with HNC suffer from sarcopenia, a condition characterized by the skeletal muscle and weight loss resulting in a decrease of physical performance.

According to some authors the prevalence of sarcopenia in patient treated by HNC is 35.5–54.5%, which is mainly attributed to weight loss and malnutrition^76,77^. Among the main mechanisms of sarcopenia include an intensification of protein catabolism probably related to tumor growths^78^.

In addition to this, CHT and RDT exacerbate this malnutrition and weight loss^79^. In some cases, CHT has a direct influence on muscle catabolism leading to a loss of muscle strength^78^, in others, the loss of muscle mass can be explained by CHT adverse effects such as fatigue, loss of appetite, nausea, vomiting or diarrhea, as a result of several reduction of food intake and physical activity^78^. When toxicities such as xerostomia, dysphagia or oral mucositis occur, a reduction of oral intake may imply malnutrition, weight loss and subsequent sarcopenia^79^. Unlike CHT, the mechanism of RDT in the onset of sarcopenia remain poorly understood. Sarcopenia is thought to decrease survival and increase disease relapses or the likelihood of death of these patients^80^.

Exercise prevents sarcopenia because it increases muscle mass and function^77,81^. Strength exercises are one of the most effective modalities to promote the upper limb functionality. However, long time exposure to exercise is required to draw muscular and neural adaptations^82^.

Given that CHT produces malnutrition due to a decreased intake and physical performance an improvement of nutritional status is mandatory^83^. Healthy lifestyle education in HNC patients encourages the acquisition of healthy feeding behavior in the long term. It is previously shown that doing exercise reduces carbohydrates and lipids intake leading to an indirect incorporation through daily meals of proteins, oligoelements and other minerals improving muscle health^84^.

Exercise-based rehabilitation increases lower limb strength in patients with HNC who had received CHT or RDT. In this sense, Lønbro et al.^58^ found that RET have a positive effect on the potentiation of lower limb muscle strength in the medium term in patients receiving CHT or RDT. As describe above a reduction of muscle strength as a result of cancer-related sarcopenia or the administration of CHT and RDT agents affect muscles equally. Thus, a RET program in the lower limb promotes the knee extension peak of force through an increase of hamstring complex muscle mass in the medium term. In this case, muscle mass changes could be associated to the fact, on the one hand, that lower limb musculature allows higher force loads compared to upper limb, and on the other hand, the higher frequency and longer duration program carried out as it has been formerly published in other populations^85,86^.

Rogers et al.^53^ also found that RET has a positive effect on the enhancement of lower limb muscle strength in the medium term in patients receiving CHT or RDT. For his part, Zhao et al.^56^ found that in HNC patients receiving CHT or RDT, the combination of RET with AET has a positive effect on the enhancement of lower limb muscle strength in the medium term. Authors differ on the effects of RET combined with AET. For some of them, hypertrophy produced by AET depends on modality, frequency, and intensity so that the higher they are the more hypertrophy there is. For many others, when they are combined, the effects can be inhibited by each other^87,88^. Nevertheless, more clinical trials are needed to delve deeper into this question.

Flexibility exercises within a multimodal program also impact on the increase of muscle maximum strength. In this sense, Lin et al.^50^ found when combining RET with AET flexibility exercises has a positive effect on the enhancement of lower limb muscle strength in the medium term in patients receiving CHT and RDT. Despite the discrepancies between some studies, recent works have shown that chronic exposure to elasticity exercises can contribute to an increase in maximum contraction force due to changes in both musculotendinous stiffness and neural impulse. It is probably due to changes in the muscle tension-length relationship and the rate of sarcomere shortening. In the case of Lin et al.^50^ active stretching exercises in combination with maximal strength exercises seem to be more effective than when they are performed with explosive force exercise, currently, not supported by evidence^89,90^.

Exercise-based rehabilitation was successful in reducing disease-related fatigue in patients with HNC. Samuel et al. found that the combination of RET with AET has a positive effect on short-term fatigue in patients receiving CHT and RDT^51^. Fatigue occurs both in the active phase of the disease and during the treatment phases producing a significant impairment of the functionality of the HNC survivor^91^. This disorder is defined as a distressing, persistent, and subjective feeling of physical, emotional, and/or cognitive tiredness or exhaustion related to cancer or cancer treatment non-proportional to activity that interferes with functioning^92^. The prevalence of fatigue in oncologic population ranges from 26.2 to 56.3% depending on the study^93^, but, in the HNC is moderate to severe only in 18% of patients^94^.

Fatigue can be caused by a set of mechanism whose etiologies are not clearly elucidated. In general, it can be attributed to both factors related to the CNS (*neuroinflammation, hypothalamic pituitary adrenal (HPA) axis*, etc*.)* and the PNS (*cachexia, alteration of energy metabolism*, etc.). It is *central* when the patient is not able to perfom physical or mental task without major cognitive or motor impairment^92^. Fatigue is peripheral if the patient’s muscles are not able to perform a task after being stimulated or there is a reduction in endurance^92^.

Both cancer and its treatment activate the inflammation cascade increasing cytokines releasing that act on the CNS. Other potential mechanism is the alteration of the HPA axis that is induced by cytokines as part of this neuroinflammation. Activation of the HPA axis increases cortisol releasing to limits systemic damages due to this inflammatory state. This will lead to physical fatigue, circadian cycle disturbances and lack of sleep^92^.

Fatigue in these patients is a result of loss of physical condition, combined with radiation and chemotherapy treatments, resulting in changes in anaerobic metabolism that lower lactate thresholds leading to fatigue and weakness. In addition, loss of appetite and the presence of nausea and vomiting can lead to caloric and nutrient deficiencies.

Fatigue-related functions may also occur at the peripheral level due to changes in energy metabolism. Not only from cachexia, but also from sarcoplasmic reticulum and/or mitochondrial damage caused by CHT and RDT, skeletal muscle may be compromised, increasing fatigue more than random or milky, depending on the primary fuel consumed^92^. The lactic acid system converts glucose into cellular energy nucleotides (AMP, ADP, ATP), which generate lactic acid as a by-product, and when lactic acid accumulates in the muscles, it produces fatigue and the patient manifests weakness, which is an illness with symptoms caused by CHT or RDT can exacerbate anorexia, nausea, or vomiting, ultimately reducing caloric intake and thus the availability of energy substrates. The lactate pathway was the predominant pathway activated when the various RETs of the program of Samuel et al. performed on HNC patients who had undergone CHT or RDT. This improvement could be explained by exercise shifting the lactate threshold to the right, leading to increased lactate tolerance in cancer patients undergoing CHT or RDT (whose threshold is generally lower) by preventing the onset of delayed fatigue^91^.

Fatigue-related functions may also occur at the peripheral level due to changes in energy metabolism. Not only from cachexia, but also from sarcoplasmic reticulum and/or mitochondrial damage caused by CHT and RDT, skeletal muscle may be compromised, increasing fatigue more than random or milky, depending on the primary fuel consumed^92^. The lactic system generates by-product lactic acid that when accumulates in the muscle generates fatigue that the patient manifests as weakness, a symptom that can worsen by CHT or RDT which lack of appetite, nausea or vomiting that end up reducing caloric intake and consequently decreasing the availability energy substrates. The lactate pathway was the predominant pathway activated when the various RETs of the program of Samuel et al.^51^ performed on HNC patients who had undergone CHT or RDT. This improvement could be explained by exercise shifting the lactate threshold to the right, leading to increased lactate tolerance in cancer patients undergoing CHT or RDT (whose threshold is generally lower) by preventing the onset of delayed fatigue^91^.

### Effectiveness of exercise-based rehabilitation in HNC survivors undergone surgery

Exercise-based rehabilitation has a positive effect on overall pain in HNC survivors undergoing surgery. In this sense, Loh et al.^65^ found that relaxation exercises have a positive short-term effect on reducing overall pain in patients undergoing head and neck surgery. As noted, apart from CHT and RDT, HNC treatment also includes surgery which appear to be effective as in early as advanced stages of the disease^18^.

Among surgical techniques, modified radical neck dissection (MRND), selective dissection of the lymph nodes of the neck, with preservation of the accessory nerve or resection of cervical nerve root branches and subsequent flap reconstruction are the most commonly used procedures^95–97^.

Neuromuscular complications due to surgical treatment are more frequent in HNC survivors compared to those who only received CHT or RDT. When cancer invades laryngopharyngeal region, musculoskeletal structures related to speech and swallowing (i.e. *dysphagia, hoarseness*, etc.) need to be removed^97,98^. Although the surgical procedure is safe, some neuromuscular complications due to sacrifice of the accessory nerve have been reported in cases related to cancers or inadvertent injury to the nerve during surgery. As a consequence, an alteration of the strength and motor coordination of the cervico-scapular musculature could develop, which could alter the functionality of the upper limb^99–101^. Another serious post-operative complication is shoulder pain which depends on the type of surgical technique performed^102,103^.

In order to relieve pain, Loh et al. found that progressive relaxation exercises have a positive effect on the reduction of overall pain in the short-term^65^. Muscle relaxation techniques improve pain in HNC undergoing surgery by decreasing anxiety and stress^104^. The mechanism that could best explain these positive effects is decreasing serum cortisol levels that has been previously associated in with the onset of myofascial pain in these subgroup cancer patients^105^. However, there is not consensus among authors about this hypothesis regarding the role of relaxation exercise on pain releasing as Kim et al. do not find blood cortisol reductions in patients with colorectal cancer^106^. Further studies are required to identify the mechanisms of the effects of exercise on postoperative pain in HNC cancer.

Overall pain in patients undergoing surgery can be reduced by joint mobility exercise in the short-term. This kind of exercise consist of the displacement of joint segments in different dimensions of space with the aim of reaching the maximum range of joint play^107^. Behind these beneficial effects on overall pain are probably the improvement on somatosensory cortical representation of movement^108^, the activation of the endogenous inhibition analgesic system^109^, and the decreasing of psychological factors related to pain (*fear-avoidance behavior*, etc.)^110,111^.

The amelioration of functionality associated with overall pain produced by the program was probably due the fact that joint mobility exercises, combined with maximum active stretching to the limit of pain tolerance, relying on through the counterirritation mechanism a phenomenon that, according to Wall and Melzack (1965), occurs as a result of introducing a noxious stimulus more intense than the base pain achieving a downward modulation of it^112^.

Also exercise-based rehabilitation has a positive effect on shoulder pain in HNC survivors who undergo surgery. McGarvey et al.^57^ found that RET, cervical mobilization exercises have a positive long-term effect on reducing shoulder pain in patients undergoing surgery. Shoulder pain is present in at least 70% of patients after non-selective dissection of the lymph nodes of the neck. Unlike radical neck resection, MRND removes all lymph nodes by radical dissection, but respects one or more of the non-lymphatic structures (*spinal accessory nerve, jugular vein, or sternocleidomastoid muscle*). In nodal selective dissection, non-lymphatic structures are preserved, and nodule removal is done according to the location of the metastases^113^.

The positive effects of the exercise program of McGarvey et al.^57^ were probably attributed to that the included participants were undergone a surgical technique with nerve root, nerve, or its spinal branches preservation, and thus, reducing the possibility of neuropathic pain linked, not only to the mechanical damage of the nerve but also with the entrapment produced by post-surgical fibrosis at the interfaces of the nerve path^57^. The positive effects of RET program on shoulder pain depend, as discussed above for CHT patients, on the increase in muscle pain threshold. Its combination with cervical mobility exercises, which the subsequent activation of endogenous pain inhibition systems, and in the study of McNeely^59^, with muscle stretching, that decreases the activation of muscle nociceptors, result in a significant improvement of shoulder pain^59,71^.

Despite not having found effects in favor of exercise on fatigue in patients with post-surgical HNC, Valkenet et al. found that inspiratory RET has a positive short-term effect on reducing fatigue in those who were operated^60^.

As commented, fatigue-related functionality is the result of a peripheral mechanism accompanied by a reduction in energy metabolism achieved initiation of the anaerobic metabolism pathway leading to fatigue of the muscles involved in ventilation. This fatigue can also be explained by the increased ventilatory rate as part of the onset of systemic inflammatory response syndrome (SIRS) due to surgery in which it is aggravated by the previous state of cancer cachexia that occurs with loss of muscle mass and loss of function^114^.

## Limitations

The methodology carried out to conduct this study implies several limitations. First, most of the RCTs include in this meta-analysis have significant heterogeneity and low sample sizes could be an important limitation for external validity of our results. Secondly, the good quality and the high risk of bias detected on the allocation concealment and the blinding of participants or therapist can produce an overestimation of the impact of exercise in the studied outcomes resulting in the possibility of misinformed conclusions. These limitations imply the need for further research into the effectiveness of standardized clinical trial protocols to refute the results and obtain more evidence to reach the level of clinical recommendation.

## Conclusions

There is evidence of fair to good methodological quality, low to moderate risk of bias, and weak recommendation supporting the use of exercise-based rehabilitation to increase functionality. However, no evidence was found in favor of the use of this modality for improving the quality of life of HNC survivors who underwent chemoradiotherapy or surgery. The lack of standardization in the development of exercise programs, the diversity of randomized trials, and the heterogeneity of interventions and evaluations warrant further study.
