# Author Correction: Exercise‑based rehabilitation on functionality and quality of life in head and neck cancer survivors. A systematic review and meta‑analysis

**DOI:** 10.1038/s41598-023-37292-w

**Published:** 2023-06-22

**Authors:** Isidro Miguel Martín Pérez, Sebastián Eustaquio Martín Pérez, Raquel Pérez García, Diego de Zárate Lupgens, Germán Barrachina Martínez, Carolina Rodríguez González, Nart Keituqwa Yáñez, Fidel Rodríguez Hernández

**Affiliations:** 1 Departamento de Medicina Física y Farmacología, Área de Radiología y Medicina Física, Facultad de Ciencias de la Salud, Universidad de la Laguna, 38200 Santa Cruz de Tenerife, Spain; 2 Escuela de Doctorado y Estudios de Posgrado, Universidad de la Laguna, 38203 San Cristóbal de La Laguna, Santa Cruz de Tenerife, Spain; 3 Musculoskeletal Pain and Motor Control Research Group, Faculty of Health Sciences, Universidad Europea de Canarias, 38300 La Orotava, Santa Cruz de Tenerife, Spain; 4 Musculoskeletal Pain and Motor Control Research Group, Faculty of Sport Sciences, Universidad Europea de Madrid, 28670 Villaviciosa de Odón, Madrid, Spain; 5 Hospital Universitario de Canarias, 38320 San Cristóbal de la Laguna, Santa Cruz de Tenerife, Spain

Correction to: *Scientific Reports* https://doi.org/10.1038/s41598-023-35503-y, Published online 26 May 2023

The original version of this Article contained an error in the spelling of the author Nart Keituqwa Yáñez, which was incorrectly given as Nart Keituqwa Yán.

The original Article has been corrected.

